# Untargeted Metabolomics and Chemometrics Elucidate Dynamic Plasma Profile Changes Induced by Cocoa Shell in Female Rats

**DOI:** 10.3390/nu17050885

**Published:** 2025-02-28

**Authors:** David Ramiro-Cortijo, Miguel Rebollo-Hernanz, Pilar Rodríguez-Rodríguez, Santiago Ruvira, Silvia M. Arribas, Maria A. Martin-Cabrejas

**Affiliations:** 1 Department of Physiology, Faculty of Medicine, Universidad Autónoma de Madrid, C/Arzobispo Morcillo 2, 28029 Madrid, Spain; david.ramiro@uam.es (D.R.-C.); pilar.rodriguezr@uam.es (P.R.-R.); santiago.ruvira@estudiante.uam.es (S.R.); 2 Food, Oxidative Stress and Cardiovascular Health (FOSCH) Research Group, Universidad Autónoma de Madrid, 28049 Madrid, Spain; miguel.rebollo@uam.es; 3 Department of Agricultural Chemistry and Food Science, Faculty of Science, Universidad Autónoma de Madrid, C/Francisco Tomás y Valiente, 7, 28049 Madrid, Spain; 4 Institute of Food Science Research (CIAL, UAM-CSIC), Universidad Autónoma de Madrid, C/Nicolás Cabrera, 9, 28049 Madrid, Spain

**Keywords:** cocoa shell extract, metabolomics, bioavailability, methylxanthines, lipid metabolism, energy homeostasis, neurotransmission, functional food, cocoa by-product

## Abstract

Objective: This study aimed to explore the effects of cocoa shell extract (CSE) supplementation on the plasma metabolome of female rats. Methods: Female rats were supplemented with CSE (250 mg/kg/day) over seven days, and plasma samples were collected at baseline, day 4, and day 7 for untargeted metabolomic profiling using LC-ESI-QTOF. Results: A total of 244 plasma metabolites were identified, while 180 were detected in the CSE. Among these, only 21 compounds were consistently detected in both the CSE and the plasma at baseline and day 7. Notably, just three compounds, caffeine, theobromine, and *N*-isovaleroylglycine, were bioavailable, detected only in plasma after supplementation on day 7, confirming their absorption and systemic distribution. Pathways related to caffeine metabolism, glycerophospholipid biosynthesis, nicotinate, and nicotinamide metabolism were significantly upregulated, indicating enhanced lipid metabolism and energy homeostasis. Conversely, reductions were observed in pathways involving tryptophan, glutathione, arginine, and proline, pointing to shifts in amino acid metabolism and antioxidant defense mechanisms. Network analysis revealed significant changes in the cholinergic synapse, retrograde endocannabinoid signaling, and glutamatergic synapse pathways, which are crucial for cellular communication and neurotransmission. Conclusions: The observed metabolic reconfiguration demonstrates CSE’s rapid modulation of the metabolome, highlighting the bioavailability of its key components. These findings suggest potential mechanisms for CSE as a functional food ingredient with health-promoting effects, potentially supporting cognitive function and metabolic health through energy metabolism, neurotransmission, and lipid signaling pathways.

## 1. Introduction

The expanding field of nutraceuticals shows an increasing interest in potential applications of food industry by-products [1]. As efforts to minimize waste and optimize resources are gaining attention, these frequently underutilized by-products are becoming recognized for their significant health-promoting properties. They represent a vast and largely unexplored resource, and up-cycling them into value-added products, such as bioactive food ingredients and nutraceuticals, not only supports sustainable development goals, but can also contribute to reducing the burden of chronic diseases [2]. Cocoa shell is an excellent example of an underutilized by-product generated during chocolate processing, usually discarded in large quantities. Recently, cocoa shell has been uncovered as a safe bioactive food ingredient, due to its rich nutritional composition and the absence of potential toxic effects [3]. Despite its classification as waste, cocoa shell is rich in bioactive compounds, such as methylxanthines, (poly)phenols, and dietary fiber, among others, which have been linked to a variety of health benefits, including antioxidant, anti-inflammatory, lipid-lowering, and vasoactive properties [4,5]. The presence of these beneficial compounds in cocoa shell has attracted considerable interest in exploring its potential role in health promotion, as it has potential in preventing diseases [6,7], particularly those associated with metabolic and cardiovascular dysregulation [8]. These diseases represent a significant global health burden, due to their high prevalence and associated mortality rates. Consequently, dietary interventions, especially those involving natural compounds, emerge as promising strategies for these health issues [9]. In this context, cocoa shell appears as a promising alternative, with the added value of representing an important milestone in the sustainable use of food by-products for health promotion.

Whereas the bioaccessibility, related colonic microbiota biotransformation, and potential absorption of methylxanthines and (poly)phenols from cocoa shell have been investigated in vitro [10], a comprehensive understanding of its bioavailability and subsequent impact on the metabolome remains unexplored. Metabolomics is a powerful tool for investigating this gap, as it allows for a comprehensive analysis of the small-molecule metabolites present in a biological system [11] and complex biochemical responses to dietary interventions, providing insights into their mechanistic foundation [12]. Untargeted metabolomics allows for a broad-spectrum view of the metabolome, identifying changes in the levels of multiple metabolites simultaneously, regardless of prior assumptions. This approach can lead to a better knowledge of the systemic effects of dietary intake, which is essential for developing effective bioactive food ingredients and nutraceuticals, advancing our comprehension of nutrition’s role in health and disease [13]. In addition, chemometric techniques can complement metabolomic analyses, and identify patterns and relationships among the complex array of plasma metabolites using advanced statistical methods, thereby highlighting the primary compounds and pathways affected by dietary interventions [14]. These observations may provide insights into the probable mechanisms through which cocoa shell exerts its beneficial effects.

Considering the limited data on the metabolic effects of cocoa shell extract, the present study aims to investigate the impact of CSE intake on the rat plasma metabolome. By employing untargeted metabolomics and chemometric analysis, we seek to identify the key metabolites and enrichment metabolic pathways influenced by cocoa shell, thereby elucidating the plasmatic changes induced by this dietary intervention. Our results could provide novel insights into the mechanisms implicated in the health-promoting properties of cocoa shell, potentially guiding its utilization as a bioactive food ingredient or nutraceutical.

## 2. Materials and Methods

### 2.1. Preparation of Cocoa Shell Extract

The cocoa (*Theobroma cacao*) shell used in this study, supplied by Chocolates Santocildes (Castrocontrigo, León, Spain), was of the high-quality “Criollo Carenero” variety from Barlovento (Barlovento, Venezuela), processed with long fermentation and low-temperature oak wood drum roasting. CSE was prepared using an optimized extraction protocol [15]. First, the cocoa shell was milled, and then the ground cocoa shell was combined with boiling water (20 g/L). The mixture was stirred continuously for 90 min, and the CSE was filtered and frozen at −20 °C for 24 h. The extract was then freeze-dried and stored at −20 °C until further use.

### 2.2. Formulation of Cocoa Shell Supplement

The CSE supplement was prepared using gelatin as a vehicle, as previously described [16]. Briefly, the gelatin cubes were formulated using 100% bovine gelatin (Inkafoods, S.L., Barcelona, Spain) dissolved in water (140 g/L). To produce the cubes, water was heated to a temperature of 50–60 °C, and the gelatin was gradually added, while stirring continuously, until it completely dissolved. At this stage, various additives were introduced into the mixture, including vanilla flavor (4.8 mL/L; MyProtein, Hut.com Ltd., Manchester, UK) and sucralose (0.6 g/L sucralin, Sucralose S.L., Barcelona, Spain) as non-caloric flavoring agent and sweetener, respectively. Two types of gelatin cubes were produced: (i) neutral cubes without CSE (vehicle) and (ii) CSE-enriched gelatins (treatment). In this case, the CSE was incorporated into the mixture after the gelatin had dissolved. The gelatin mixture was then carefully poured into a mold, ensuring even distribution, to form cubes with a size of 1 cm^3^. The dose of CSE used in the study was 250 mg/kg/day, which was calculated based on the rat’s weight and the cube’s size. A comprehensive flow chart of the experimental design is depicted inFigure 1.

### 2.3. Protocol for Cocoa Shell Supplementation in Female Rats

Five-month-old adult female Sprague Dawley rats from the breeding colony at the animal house facility of Universidad Autónoma de Madrid (ES-28079-0000097) were utilized for the study. The experimental procedures adhered to the Guidelines for the Care and Use of Laboratory Animals (National Institutes of Health publication no. 85–23, revised in 1996), Spanish legislation (RD 53/2013), and the Directive 2010/63/EU on the protection of animals. Ethical approval was obtained from the Ethics Review Board of Universidad Autónoma de Madrid and the Regional Committee of Comunidad Autónoma de Madrid (PROEX 19/04; approval date: 20 March 2019). The rats were group-housed in type III cages (24 cm × 19 cm × 45 cm; length × height × width) with poplar bedding, accommodating 3–5 rats per cage. Environmental enrichment was provided using cellulose nestles and play tunnels (Index Research S.L., Madrid, Spain). The animals were maintained under controlled temperature (22 °C), humidity (40%), and a 12 h light–dark cycle. They were fed ad libitum with a diet containing 51.7% carbohydrates, 21.4% protein, 5.1% lipids, 3.9% fiber, 5.7% minerals, and 12.2% humidity (SafeA03; Safe-Lab, Augy, France). Drinking water was also available ad libitum. Training staff regularly monitored animal health to ensure that the rats were free from any pathogens that could influence the study parameters.

CSE supplementation was performed through voluntary ingestion. The rats were first trained to accept the new food using neutral gelatin cubes for 3–5 days, following a previously established protocol [16]. After the training phase, the rats were supplemented with CSE-enriched gelatin cubes for a period of 7 days. Blood samples were collected from the rats on day 0 (baseline, non-supplemented), day 4, and day 7. The blood samples were collected in restrained rats through the tail vein, using tubes preloaded with 5% heparin. The blood was centrifuged at 900× *g* for 10 min at 4 °C, and the plasma was aliquoted and stored at −80 °C for further analysis. The rats were not sacrificed as part of this specific study. However, they were used in a subsequent study, after which they were humanely euthanized with CO_2_ exposure, followed by exsanguination [16].

### 2.4. Sample Preparation for Untargeted Metabolomic Analysis

#### 2.4.1. Preparation of CSE

CSE was weighed (10 mg) and added to a 2 mL tube. Then, 1 mL of MeOH:H_2_O (80:20 *v*/*v*) was added to the tube. The tube was placed in a Thermomixer and agitated at 2000 rpm, for 2 min, at a temperature of 4 °C. Subsequently, the tube was subjected to cold sonication for 10 min. After sonication, the tube was centrifuged at 12,000× *g*, for 10 min, at a low temperature, and the supernatant was collected without disturbing any solid particles settled at the bottom of the vial. This supernatant was then suitable for injection. A blank extraction sample was also prepared, following the same extraction protocol used for the experimental samples to ensure accuracy and control for potential interferences.

#### 2.4.2. Preparation of Plasma Samples

Samples were thawed on ice and homogenized using a vortex for 30 s. Then, 200 µL of plasma was mixed with 800 µL of MeOH, previously cooled to −20 °C. The mixture was vortexed for 2 min and incubated at −20 °C for 1 h. Subsequently, the samples were centrifuged at 12,000× *g* for 15 min at 4 °C. The supernatant (600 µL) was collected without disturbing the solid particles at the bottom of the vial. The supernatant was dried using a Speed-vac for 5 h at room temperature. For reconstitution, 200 µL of MeOH:H_2_O (80:20 *v*/*v*) was added, followed by vortexing for 2 min and 30 s. The reconstituted solution was centrifuged at 12,000× *g* for 10 min at 4 °C, and the supernatant was collected, without disturbing any solid particles at the bottom of the vial, for injection. A blank extraction sample was prepared, following the same extraction protocol as the experimental samples.

### 2.5. Untargeted Metabolomic Analysis by LC-ESI-QTOF

An Agilent 1290 Infinity UHPLC system (Santa Clara, CA, USA) was used for metabolomic analysis, equipped with a binary pump, a diode array detector, and a Peltier-cooled autoinjector. This system combined ultra-high pressures (up to 1200 bar) with high mobile-phase flow rates (up to 5 mL/min). The exact mass spectrometer used was the Agilent 6540 UHD (Santa Clara, CA, USA), with a quadrupole time-of-flight (Q/TOF) analyzer and an ESI Jet Stream interface (Santa Clara, CA, USA). The column used was a Zorbax Eclipse Plus C18 (1.8 µm, 2.1 × 100 mm), coupled with a Zorbax C18 precolumn (1.8 µm, 2.1 × 5 mm) from Agilent (Santa Clara, CA, USA). The column temperature was maintained at 40 °C, and the injection volume was set to 2 µL. The mobile phase consisted of phase A (0.1% formic acid in H_2_O) and phase B (0.1% formic acid in acetonitrile), with a 0.5 mL/min flow rate. The gradient started with 0% of the organic mobile phase B (0.1% formic acid in acetonitrile) at the beginning of the analysis, and it increased linearly to 100% over 13 min. At 14 min, the gradient rapidly changed to 0% organic mobile phase, and this composition was maintained for 3 min for column equilibration before the next injection. Detection was performed using TOF-MS in positive mode, with a mass range of 25–1100 *m*/*z* and a scan rate of 5 spectra/s. The ionization source was AJS-ESI, and the gas temperature was 300 °C. The drying gas flow rate was 8 L/min, the nebulizer pressure was 40 Psi, and the sheath gas temperature was 350 °C, with a flow rate of 11 L/min. The capillary voltage was set at 3000 V, and the fragmentor and skimmer voltages were 110 V and 45 V, respectively. Reference masses for calibration were *m*/*z* 121.050873 (purine) and 922.009798 (HP-0921). The same TOF conditions were applied for Q-TOF MS/MS analysis in the positive mode. Collision energies (CID) of 20 and 40 were used for fragmentation studies. The samples were injected randomly to eliminate any drift effects that could arise from the instrument or analysis conditions. Blank injections were intercalated after each sample injection to monitor the proper elution of sample components, prevent sample contamination (carry-over), and improve reproducibility. Three replicates were performed for each sample and blank extraction, with a blank injection (H_2_O MilliQ) between each replicate.

### 2.6. Data Statistical Analysis

#### 2.6.1. Data Curation and Processing

The chromatograms acquired were processed using MS-DIAL software (version 4.6) (http://prime.psc.riken.jp/compms/msdial/main.html (accessed on 19 September 2024)). All the sample and extraction blank files (.d) were converted to .abf format and simultaneously analyzed. Peak detection was performed using the retention time and the exact mass, and the MS2Dec deconvolution algorithm was utilized. This algorithm initially extracts the MS/MS spectra for each precursor peak across all chromatograms, then employs least squares optimization to extract the “model peaks”. Finally, the pure MS/MS spectrum is determined by the maximum heights of the reconstructed chromatograms. Peak alignment was subsequently carried out, and compound identification was performed using the exact mass, isotope ratio, and MS/MS spectrum similarity, by comparison with various databases (NIST20, MoNA, and LipidBlast).

For post-processing of the data, the median of the heights of each triplicate injection was considered. A series of filtering steps were then conducted. This involved eliminating all peaks whose maximum height in the samples was less than three times the average height of the peak in the extraction blanks. Furthermore, all peaks with a maximum height in the samples of less than 1000 intensity units were excluded. In addition, all peaks not identified as metabolites in the matched libraries were discarded, along with any peaks not quantified in at least three samples from any group. According to the PubChem repository (https://pubchem.ncbi.nlm.nih.gov/), each peak was identified with the InChIKey code. Subsequently, the low limit of detection (LLD) was considered as the lowest level of intensity in the identified metabolite. To avoid artifacts in the statistical analysis, the non-detected intensity of metabolite was filled with ½ of LLD. Finally, duplicates were eliminated, and the adducts and fragments found for the same metabolite were grouped using the bioinformatics tool MS-FLO (https://msflo.fiehnlab.ucdavis.edu/). This comprehensive data curation process ensured the accuracy and reliability of the subsequent metabolomic analysis.

#### 2.6.2. Univariate Statistical Analysis

The metabolomic analysis followed the workflow described in Chen et al. [17]. The analysis was performed by R software version 4.4.1 (R Core Team 2022. R Foundation for Statistical Computing, Vienna, Austria; https://www.R-project.org/ (accessed on 3 July 2024) with the RStudio interface (version 2023.06.0+421 for Windows; Boston, MA, USA). Overall, the packages used were *rio*, *dplyr*, *compareGroups*, *ggplot2*, *ggpubr*, *grid*, and *gridExtra*; the specified packages are described below. A *p*-value of less than 0.05 was considered statistically significant in all analyses. The distribution of each metabolite was examined using the Shapiro–Wilk test, to ensure that the subsequent analysis was applicable. The metabolite variables were logarithmically converted and reported as medians and interquartile ranges.

The categorical variables were summarized as relative frequencies. Univariate analysis was used to identify differences in the abundance of the metabolites over time. Considering the same individual, a repeated Mann–Whitney test was performed when day 4 was excluded. In addition, the *p*-value was adjusted for multiple comparisons by false discovery rate (FDR).

#### 2.6.3. Multivariate Chemometric Analysis

This analysis was performed using Principal Component Analysis (PCA), subclass fold change, and heat maps of metabolites by time points. This analysis was carried out by the *omu* [18], *pheatmap*, *FactoMineR* [19], and *factoextra* packages. Firstly, the metabolomic data were normalized by typification and scaled between −1 and 1. This step was essential to ensure that all variables were on a comparable scale, avoiding undue influence from variables with large numeric ranges. Secondly, unsupervised PCA was carried out to capture the maximum variance in the dataset, by reducing its dimensionality while preserving the essential similarities and differences between the samples. The analysis was performed simultaneously on all samples for the three replicates, to identify patterns and visualize clustering. The PCA was performed following the sphericity assumption based on Barlett´s test. To avoid overlapping in the metabolomic variables, the standardized loading was extracted from the varimax-rotated matrix, and each sample’s weight in the first and second principal components (PCs) was reported.

In addition, heatmap and dendrogram analyses were performed to classify the samples by time. This visual approach allows an intuitive understanding of the relationships among the samples based on their metabolic profiles, highlighting the distinct clusters within the data, and, thus, corroborating the findings from the PCA and fold change analysis. The metabolomic data were classified according to Euclidian distance and clustered by the Ward method. For an appropriate interpretation, the metabolic variables were split according to significant differences in the fold change for their subclass.

#### 2.6.4. Pathway and Enrichment Analysis

The InChIKey codes were matched with their Human Metabolome Database (HMDB) ID, Kyoto Encyclopedia of Genes and Genomes (KEGG) ID, and PubChem Compound Identification (CID) using a chemical translation service (http://cts.fiehnlab.ucdavis.edu/ (accessed on 19 September 2024)) [20]. All metabolites had a PubChem CID, but not all metabolites were identified by the HMDB and KEGG, because metadata were unavailable for some. Then, the pathway and enrichment analyses were performed by the MetaboAnalyst 5.0 platform (https://www.metaboanalyst.ca/MetaboAnalyst/ (accessed on 19 September 2024)). The metabolites identified were contrasted with the pathways available in all libraries for the *Rattus norvegicus* model, using relative-betweenness centrality in the topology analysis and hypergeometric test. For the pathway analysis, the pathway impact was calculated as the sum of the importance measures of the matched metabolites divided by the sum of the importance measures of all metabolites in each pathway, and the logarithmic *p*-value was extracted. In addition, for the enrichment analysis, the metabolites were clustered by subclass of chemical structure, and the enrichment ratio was computed as the observed hits introduced as metabolites divided by the expected hits of the pathway. Both the logarithmic *p*-value transformed and the FDR-adjusted *p*-value were extracted and plotted.

#### 2.6.5. Functional Analysis of Metabolic Changes

The functional analysis was conducted with a multivariate strategy to identify the major drivers of differences between time and visualize complex patterns in the metabolomic data. Then, the InChIKey codes were clustered, extracting subclasses of compounds according to the PubChem repository registered in the MeSH Three classification, LIPID MAPS Classification, or KEGG: Metabolite classification. Metabolites not found in any of the libraries were categorized as “*unknown*”. The fold change at the end of the cocoa shell supplementation (day 7) was compared to the baseline (basal time). Significant fold changes in the subclasses, adjusted for FDR, were then extracted.

#### 2.6.6. Functional Enrichment Through Network-Based Analysis

To elucidate the functional implications of our metabolomic data, we utilized the FELLA (Functional Enrichment analysis using Latent variable models for Metabolomics data) package in R [21]. The analysis aimed to integrate metabolomic data with KEGG pathway information to identify enriched pathways and key metabolites. The input for this analysis comprised significantly modified metabolites, with significant fold changes adjusted for FDR, compared to the baseline. The KEGG data were loaded from a pre-constructed local database encompassing pathway, enzyme, reaction, compound, and module information. Identified metabolites were mapped onto the KEGG graph to perform the functional enrichment analysis, prioritizing metabolites and pathways based on their relevance in the metabolic network. The diffusion method was applied with a set number of 100 iterations to ensure robust results. The results were visualized by generating a network graph using FELLA. The top-scoring nodes were determined based on a stringent *nlimit* parameter set to 150, and enriched pathways were exported for further analysis. This approach allowed for the effective integration and interpretation of metabolomic data within the context of established metabolic pathways, highlighting critical areas for subsequent investigation.

## 3. Results

### 3.1. Comprehensive Analysis of CSE Showed Key Metabolic Pathways and Chemical Structures

The comprehensive metabolomic analysis of the CSE identified a total of 180 compounds, highlighting the complexity of the extract’s chemical profile (Table 1). Detailed data on retention times, molecular formulae, ion adducts, and mass-to-charge ratios (*m*/*z*) with associated errors in parts per million (ppm) underline the robustness of the analysis. To better contextualize these findings, we have provided an expanded supplementary table (Supplementary Table S1) listing the major compounds identified, their putative metabolic pathways, and their potential health effects. Given the untargeted nature of this metabolomic study, absolute quantification is not available. However, semi-quantitative abundance counts allow for relative comparisons among metabolites. The analysis revealed a wide array of phytochemicals and bioactive compounds, reflecting the diverse chemical nature of the CSE. After adjusting for FDR, the predominant chemical structures identified were several key classes of compounds, particularly amino acids and xanthines. Among the amino acids, phenylalanine ([M+H]^+^ = 166.08574 *m*/*z*) and betaine ([M+H]^+^ = 118.08614 *m*/*z*) were highly represented, reflecting their significant roles in protein synthesis and methylation processes.

The xanthines identified, mainly caffeine ([M+H]^+^ = 195.08735 *m*/*z*) and theobromine ([M+H]^+^ = 181.07198 *m*/*z*), are well-known for their roles in central nervous system stimulation, neurotransmitter regulation, and cognitive enhancement. Both compounds are also implicated in energy metabolism regulation, reinforcing the potential metabolic benefits of CSE. The presence of these compounds suggests both neurological and metabolic benefits, potentially contributing to enhanced cognitive function and metabolic activity. Additionally, other significant compounds included phenylethylamines (known for their stimulant and psychoactive properties, influencing mood and focus), pyridine carboxylic acids (which play important roles in vitamin B metabolism and overall energy production), dipeptides (such as Val-Val and Ile-Phe, which are involved in protein synthesis, cellular repair, and metabolism), amino fatty acids (important in maintaining membrane structure and signaling pathways), and carnitines (crucial for fatty acid metabolism and mitochondrial function, particularly in energy production through β-oxidation).

**Figure 2:**
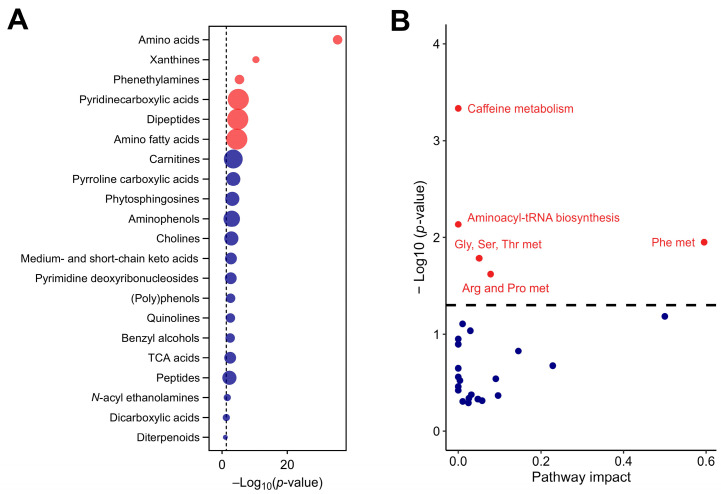
The chemical and functional composition of the cocoa shell extract (CSE), including metabolic pathways with significant impact, as identified by untargeted metabolomics (**A**) and enrichment analysis of subclass chemical structures (**B**), where the size of the dots indicates the enrichment ratio. Red dots indicate structures and pathways with significant differences, with *p*-values adjusted by the false discovery rate (FDR). Blue dots represent structures and pathways without significant differences. Gly, Ser, Thr met: glycine, serine, and threonine metabolism; Phe met: phenylalanine metabolism; Arg and Pro met: arginine and proline metabolism.

The analysis also detected pyrroline carboxylic acids (linked to the regulation of oxidative stress), quinolines (involved in various enzyme systems and redox reactions), and phytosphingosines (key mediators in lipid signaling pathways and cellular membrane stability). The detection of these compounds underlines the extract’s potential to modulate oxidative stress responses and lipid homeostasis, further emphasizing its bioactive potential. These compounds, shown in Figure 2A, contribute to the rich biochemical diversity of CSE, and suggest multiple physiological roles that extend beyond primary metabolism. The metabolic pathway analysis revealed the significant involvement of key biochemical pathways, particularly those pathways associated with phenylalanine, caffeine, aminoacyl-tRNA biosynthesis, glycine, serine and threonine, and arginine and proline metabolism (Figure 2B).

**Figure 3:**
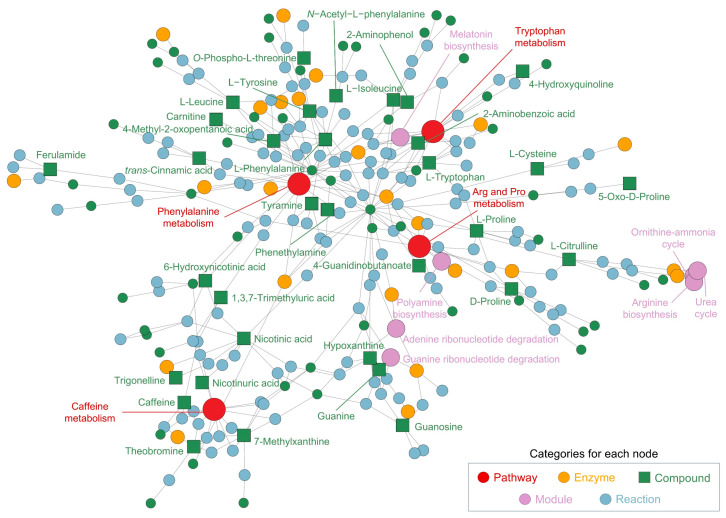
Network and pathway analysis of metabolites identified in the cocoa shell extract (CSE), illustrating the interconnected metabolic pathways and compounds. The network highlights significant metabolic pathways, including phenylalanine metabolism, arginine and proline metabolism, caffeine metabolism, and tryptophan metabolism. Nodes represent different categories: pathways (red, ●), enzymes (yellow, ●), compounds (green, ■), modules (purple, ●), and reactions (blue, ●). Arg and Pro metabolism: arginine and proline metabolism.

In addition to the pathway analysis, the network analysis (Figure 3) further elucidates the interconnections between metabolic pathways and individual metabolites identified in the CSE, offering insights into its potential bioactive applications. The network map underscores the central role of several key pathways, with phenylalanine metabolism (*p* = 0.013) emerging as a core node. Phenylalanine and its downstream products, such as L-tyrosine and tyramine, are involved in the biosynthesis of neurotransmitters, particularly dopamine and norepinephrine, which are critical for cognitive functions such as mood regulation and mental alertness. This finding suggests that phenylalanine-related metabolites in CSE could confer neuroactive properties, potentially enhancing cognitive performance and alertness.

Arginine and proline metabolism (*p* = 0.005) plays another critical role in the network, encompassing key metabolites like L-proline, L-citrulline, and L-arginine. These compounds are integral to protein synthesis, nitric oxide production, and the plant urea cycle. In humans, their well-known functions in vascular health, muscle metabolism, and recovery highlight the potential for CSE to be used in cardiovascular support and muscle recovery nutraceutical formulations. Notably, L-citrulline is associated with improved blood flow through its vasodilatory effects, underscoring the cardiovascular benefits that could be exploited from CSE-based products.

Caffeine metabolism (*p* = 0.019) also forms a prominent and interconnected cluster, including caffeine, theobromine, and 7-methylxanthine. In *Theobroma cacao*, these xanthines contribute to plant defense and growth regulation, while in humans, they are recognized for their stimulatory effects on the central nervous system. Their potential to enhance cognitive alertness and physical endurance through mechanisms such as adenosine receptor inhibition and dopamine stimulation positions CSE as a promising natural source of energy-boosting nutraceuticals. The synergy between these xanthines could further amplify these stimulatory effects, reinforcing CSE’s multifaceted role in enhancing cognitive and physical performance.

Other significant pathways include amino acid metabolism, such as glycine, serine, and threonine biosynthesis (*p* = 0.041), which is essential for protein turnover and cellular regeneration. The clustering of aminoacyl-tRNA biosynthesis within the network highlights CSE’s potential to support protein synthesis, making it beneficial for muscle health and recovery. Additionally, pyridine carboxylic acid metabolism, including nicotinic acid and 6-hydroxynicotinic acid, suggests involvement in vitamin B3 metabolism (*p* = 0.039), a crucial process for maintaining cellular energy homeostasis and lipid metabolism. This connection indicates that CSE may help to support energy production and reduce oxidative stress. Moreover, the presence of quinoline derivatives and phytosphingosines in the network suggests additional layers of bioactivity. Quinoline derivatives, for instance, have been linked to enzyme regulation and antimicrobial activity, whereas phytosphingosines are involved in lipid signaling pathways crucial for maintaining skin barrier integrity. This further extends the possible applications of CSE beyond cognitive and cardiovascular benefits, potentially opening avenues for its use in cosmeceuticals or skincare formulations aimed at enhancing skin health. The metabolic pathways associated with caffeine metabolism, purine degradation (*p* = 0.003), and guanine ribonucleotide degradation (*p* = 7.58 × 10^−5^) further reveal CSE’s role in energy and nucleotide metabolism, which are essential for maintaining cellular energy levels, proliferation, and overall metabolic balance. The creatine pathway (*p* = 1.0 × 10^−6^), essential for energy storage in muscle and brain tissues, highlights another potential use of CSE in performance-enhancing supplements or recovery aids. Furthermore, ceramide (*p* = 0.028) and sphingosine (*p* = 0.026) biosynthesis pathways indicate CSE’s potential to regulate lipid metabolism and support skin health, reinforcing its bioactive versatility.

Altogether, this comprehensive network of metabolic pathways and compounds in cocoa shell points to various potential health benefits, from neurotransmitter regulation and cardiovascular support, to skin health and metabolic balance. The compounds and pathways identified in the network underscore the multifunctional nature of CSE, supporting its use in a diverse range of nutraceutical products aimed at enhancing cognitive performance, cardiovascular health, muscle recovery, and general metabolic well-being. By leveraging the interconnected nature of these bioactive compounds, CSE could offer synergistic benefits, making it a highly versatile ingredient for nutraceutical and cosmeceutical formulations. Therefore, the metabolomic profiling of CSE provides a comprehensive overview of its chemical composition, revealing a variety of bioactive compounds with potential physiological effects. Integrating both pathway and network analysis further emphasizes CSE’s potential for having significant impacts on neurological, cardiovascular, and metabolic processes, supporting its potential use in nutraceutical formulations targeting cognitive function, energy metabolism, and vascular health.

### 3.2. Chemometric Analysis of Rat Plasma Metabolome Revealed Distinct Metabolic Profiles During CSE Supplementation

**Figure 4:**
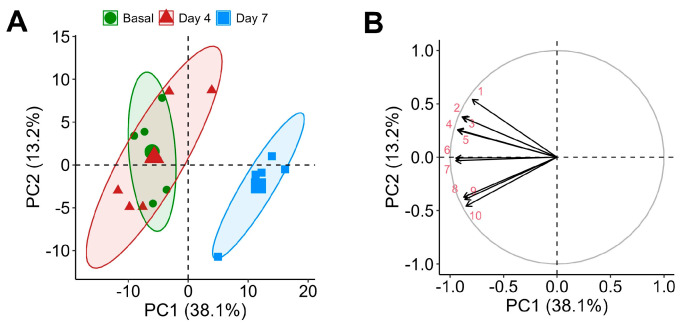
Principal Component Analysis (PCA) of the plasma metabolome with cocoa shell extract (CSE) supplementation, showing a PCA scores plot illustrating the separation of samples by time points: baseline (green circles, ●), day 4 (red triangles, ▲), and day 7 (blue squares, ■) (**A**), and a loadings plot highlighting the main metabolites contributing to the variance (**B**). Principal components (PCs): 1: ceramide 8:0;2O/14:0; 2: 12-(3-(Adamantan-1-yl)ureido)dodecanoic acid; 3: 6-Hydroxy-5a-methyl-3,9-dimethylidenedecahydronaphtho[1,2-b]furan-2(3h)-one; 4: L-Leucyl-L-alanine; 5: Octapamine; 6: 1-Myristoyl-sn-glycero-3-phosphocholine; 7: Monoacylglycerophosphocholines 18:3; 8: 1-alkyl,2-acylglycerophosphocholines 20:4; 9: 1-alkyl,2-acylglycerophosphocholines 16:0; 10: Monoacylglycerophosphocholines 22:6.

Throughout the supplementation period, a total of 244 metabolites were detected in rat plasma. Of these, 84.8% were detected at baseline, 91.8% on day 4, and notably fewer, 68.0%, on day 7, indicating dynamic metabolic adjustments throughout supplementation. The chemometric analysis of the untargeted metabolomic data revealed distinct changes in the rat plasma metabolome following CSE supplementation. PCA indicated that components 1 and 2 accounted for 51.3% of the total variance, with a discernible clustering of metabolites, denoting significant metabolic shifts. Specifically, samples from the baseline and day 4 time points were grouped closely, while those from day 7 formed a distinct cluster, demonstrating a marked shift in the metabolic profile by this time point (Figure 4A).

This clustering indicates a time-dependent metabolic response to CSE supplementation. The 10 uppermost influential metabolites in the PCA were mainly lipids, including contributions from ceramides (ceramide 8:0;2O/14:0) and glycerophosphocholine derivatives (1-myristoyl-sn-glycero-3-phosphocholine, LPC 18:3, LPC 22:6, PC O-20:4, PC O-16:0), reflecting significant lipidomic alterations, which may influence changes in membrane fluidity, signaling, or energy metabolism. Additionally, metabolites like 12-(3-(adamantan-1-yl)ureido)dodecanoic acid and octapamine were highlighted, underscoring potential modifications in fatty acid metabolism and alteration of neurotransmitter precursor levels, respectively. Other compounds contributing to the variance included 6-hydroxy-5a-methyl-3,9-dimethylidenedecahydronaphtho[1,2-b]furan-2(3h)-one, a soluble epoxide hydrolase enzyme inhibitor (a compound potentially linked to (poly)phenol metabolism and associated with antioxidant properties), and the dipeptide L-leucyl-L-alanine (indicative of altered peptide metabolism) (Figure 4B). These findings suggest that lipid and amino acid metabolism is particularly responsive to CSE supplementation.

**Figure 5:**
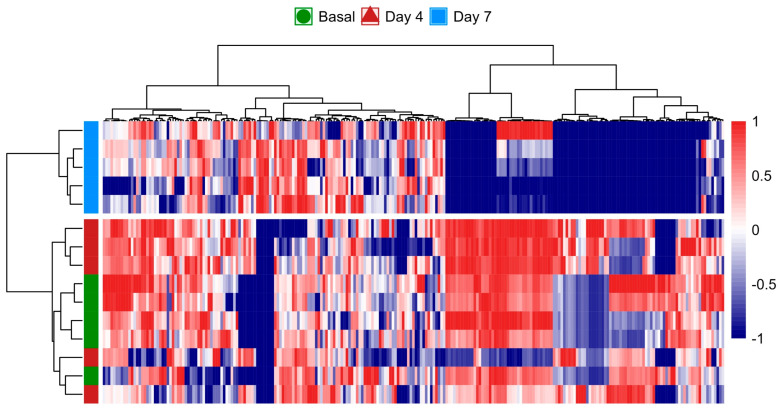
Heatmap analysis of the rat plasma metabolome over the 7-day CSE supplementation period, illustrating the progressive reconfiguration of metabolic profiles. The color scale from blue to red represents the relative intensity of metabolite levels, with blue indicating lower levels and red indicating higher levels. Hierarchical clustering on both axes shows the relationships and grouping patterns among the metabolites and time points, highlighting significant metabolic shifts by day 7.

The heatmap analysis further illustrated the temporal changes in the metabolome, where the metabolic fingerprint at the basal time point displayed only subtle differences compared to day 4. However, a pronounced divergence emerged by day 7, indicating that significant metabolic shifts had occurred over the course of the 7-day CSE supplementation (Figure 5). These shifts are visually represented by the increasing intensity of red and blue signals, especially by day 7, reflecting upregulated and downregulated metabolites, respectively. This observation suggests that the rat metabolome underwent a progressive reconfiguration over time, with minimal metabolic perturbations during the first 4 days of CSE exposure. The distinct clustering of samples from day 7 highlights a clear separation from both the basal and day 4 samples. This temporal progression implies that longer exposure to CSE is required to induce significant metabolic alterations. While the early metabolic response (day 4) appears to be more similar to the basal state, it is at day 7 that the most pronounced metabolic changes are observed. As a result, the data from day 4 were considered not substantially different from the baseline, and were subsequently excluded from further differential analysis. The heatmap reveals that several metabolites showed consistent changes by day 7, indicated by the tightly grouped red and blue patterns, suggesting potential biomarkers or key metabolites influenced by the CSE supplementation.

These alterations provide insights into the time-dependent effects of CSE on the metabolic profile, showing that prolonged exposure is necessary to elicit significant biological responses. The hierarchical clustering of the samples also demonstrates that metabolic responses were relatively consistent within each time point (day 0, day 4, and day 7). However, the most substantial metabolic deviation occurs between day 7 and the earlier time points, confirming CSE’s delayed but significant metabolic impact.

**Figure 6:**
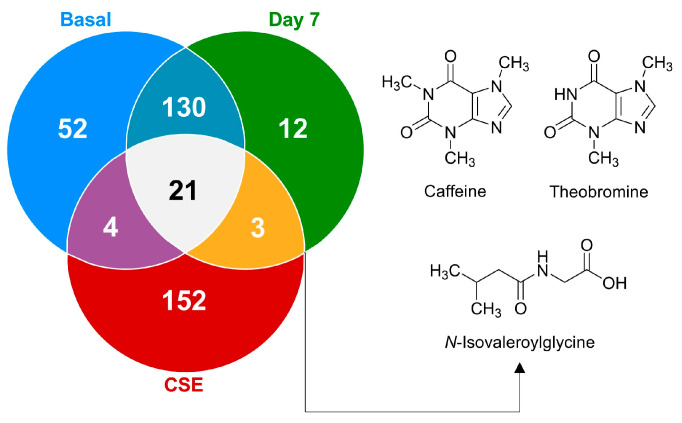
A Venn diagram illustrating the distribution of shared and unique metabolites at the basal time point, on day 7, and after cocoa shell extract (CSE) administration. The metabolites present on day 7 but absent at basal are depicted, highlighting their potential as bioactive compounds derived from CSE.

To further explore the metabolic changes and bioavailability of compounds introduced through CSE, the Venn diagram (Figure 6) illustrates the distribution of these metabolites, highlighting how the CSE contributes a unique set of compounds to the plasma after supplementation. These findings emphasize the importance of understanding the metabolites present in dietary supplements, their bioavailability, and their potential physiological relevance. The chemometric analysis underscores that while many metabolites in CSE do not directly appear in plasma, those that are bioavailable could play critical roles in driving the supplement’s health benefits. Interestingly, a significant portion of the metabolites identified in CSE (84.2%) were unique to the extract and undetectable in plasma both before and after supplementation. This suggests that many of the CSE’s components were either not absorbed or rapidly metabolized into other compounds post-ingestion. Understanding bioavailability (the extent and rate at which ingested compounds enter the systemic circulation and are available for biological activity) is essential when evaluating the efficacy of dietary supplements, as only bioavailable compounds can exert physiological effects. From the total metabolites identified, only 21 compounds (5.6%) were consistently found across all groups: the CSE, the plasma at baseline (basal), and after 7 days of supplementation (day 7). This small subset of metabolites suggests that only a limited fraction of the compounds present in the CSE circulate consistently in the bloodstream, both before and after supplementation. Among these, only three compounds (0.8% of all compounds found, 1.7% of CSE’s metabolites) were bioavailable after supplementation, being absent in the basal state but detectable on day 7. These bioavailable compounds, *N*-isovaleroylglycine ([M+H]^+^ = 160.09671 *m*/*z*), caffeine ([M+H]^+^ = 195.08735 *m*/*z*), and theobromine ([M+H]^+^ = 181.07198 *m*/*z*), are of particular interest because of their known physiological effects. Caffeine and theobromine, two well-known stimulants, are associated with increased alertness, cognitive performance, and physical endurance. Meanwhile, *N*-isovaleroylglycine, a lesser-known metabolite, has been linked to metabolic processes, suggesting potential impacts on amino acid and protein metabolism. Although the number of bioavailable compounds is relatively low, their physiological implications could be significant, particularly in enhancing cognitive function and energy metabolism. The Venn diagram (Figure 6) also shows that 130 compounds (34.8%) were shared between the basal state and day 7, suggesting that these metabolites are endogenous and unaffected by CSE supplementation. The presence of 12 unique compounds (3.2%) detectable only on day 7, and 52 compounds (13.9%) unique to the basal state, further underscores the dynamic nature of the plasma metabolome and the metabolic shifts induced by CSE supplementation. These findings highlight the importance of the bioavailable compounds and the broader metabolic changes driven by CSE. The chemometric analysis revealed that CSE supplementation induced dynamic shifts, particularly in lipid and amino acid metabolism, with the bioavailability of key compounds like caffeine and theobromine standing out. This underscores CSE’s potential physiological impacts, particularly in cognitive enhancement and energy metabolism, while also pointing to areas where CSE’s influence may remain undetected due to rapid metabolism or limited absorption.

### 3.3. Dynamic Modulation of Metabolic Pathways Induced by CSE Supplementation Was Observed by Pathway Analysis

**Figure 7:**
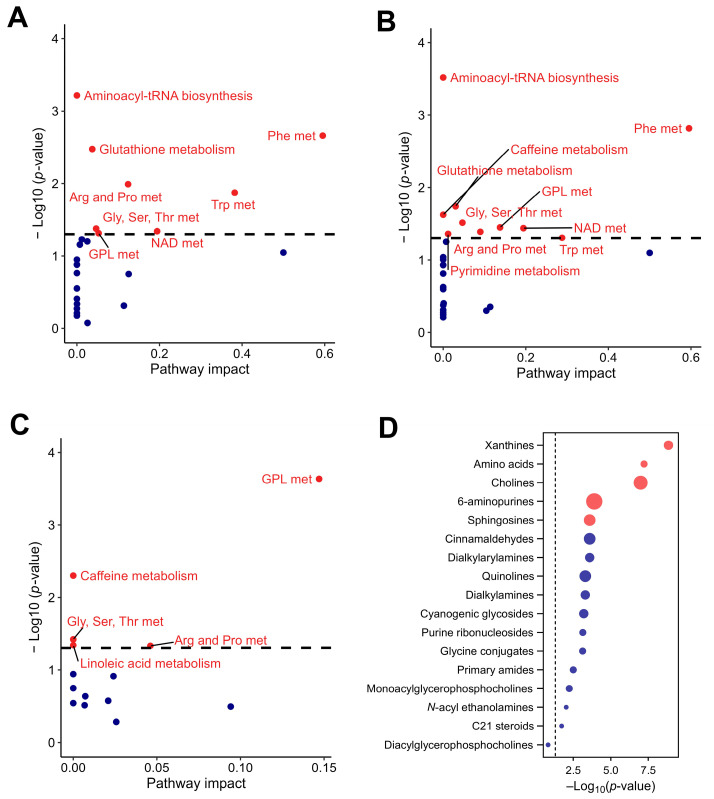
Pathway analysis of the rat plasma metabolome in response to CSE supplementation, including the metabolic pathways with significant impact at the basal time point (**A**), the metabolic pathways with significant impact at day 7 of CSE supplementation (**B**), the rat plasma pathways showing significant fold changes during the supplementation period (**C**), and a dot plot of the subclasses of chemical structures showing significant enrichment ratios (**D**). Red dots indicate pathways with significant differences, with *p*-values adjusted by false discovery rate (FDR), while blue dots indicate pathways without significant differences. Gly, Ser, Thr met: glycine, serine, and threonine metabolism; Phe met: phenylalanine metabolism; NAD met: nicotinate and nicotinamide metabolism; Arg and Pro met: arginine and proline metabolism; Trp met: tryptophan metabolism; GLP met: glycerphospholipid metabolism.

Initially, 23 signaling pathways were identified at the basal time point, which increased slightly to 24 by day 7. At the basal time point, the main pathways with significant impact were those related to the metabolism of phenylalanine, tryptophan, aminoacyl-tRNA biosynthesis, glutathione, arginine and proline, nicotinate and nicotinamide, glycine, serine and threonine, and glycerophospholipid (Figure 7A). By day 7, the metabolic pathways that maintained their impact were those related to phenylalanine, aminoacyl-tRNA biosynthesis, and glycine, serine, and threonine. These pathways are essential for protein synthesis and overall cellular function. Those that had increased impact were related to glycerophospholipid, nicotinate, and nicotinamide metabolism, suggesting augmented lipid remodeling and energy homeostasis processes. Conversely, the pathways that showed decreased impact were tryptophan, glutathione, arginine, and proline metabolism, indicating changes in amino acid metabolism and cellular antioxidant capacity. Additionally, new metabolic activity was detected in the pyrimidine and caffeine metabolism pathways, suggesting the introduction of CSE components into the host metabolism (Figure 7B).

The semi-quantitative changes revealed that 74 out of 244 metabolites showed a significant fold change from basal to day 7. The majority (58 metabolites) exhibited a decrease, while 16 showed an increase (Table 2). Supplementary Table S2 provides the key metabolites detected in rat plasma after cocoa shell extract supplementation, their associated metabolic pathways, and their potential health effects. Certain metabolites stood out due to their significant fold changes among the dynamic alterations reported in metabolic pathways after CSE administration. The 3.5-fold drop in levels of docosahexaenoic acid (DHA) methyl ester ([M+H]^+^ = 343.26376 *m*/*z*), and the 635.1-fold drop in PC O-20:5 ([M+H]^+^ = 522.35655 *m*/*z*), a phosphatidylcholine-containing eicosapentaenoic acid (EPA), might indicate higher usage or altered metabolism of omega-3 fatty acids, which are essential for brain function and have anti-inflammatory effects. Compounds related to cellular maintenance and stress response also showed significant decreases; spermidine ([M+NH_4_]^+^ = 146.16516 *m*/*z*) decreased by 2.7-fold, potentially impacting cellular proliferation and longevity, while corticosterone ([M+Na]^+^ = 347.22122 *m*/*z*) decreased by 3.0-fold, indicating a stress axis modulation. Similarly, a 2.8-fold drop in uric acid ([M+H]^+^ = 169.03545 *m*/*z*) might reflect changes in oxidative stress management and purine breakdown. Moreover, decreases of 1.4- and 1.5-fold in tyrosine ([M+H]^+^ = 182.08156 *m*/*z*) and creatine ([M+NH_4_]^+^ = 132.07704 *m*/*z*), respectively, both of which are required for neurotransmitter generation and energy storage, may indicate changes in cognitive functioning and energy dynamics. In contrast, significant increases in metabolites such as *N*-[4-(methylthio)phenyl]-*N*′-phenylurea ([M+Na]^+^ = 517.17255 *m*/*z*), which increased by 21.5-fold, and LPC O-13:1 ([M+H]^+^ = 438.29791 *m*/*z*), which increased by 7.5-fold, indicate activation of detoxification mechanisms and changes in cell membrane dynamics. Furthermore, a 2.5-fold increase in caffeine ([M+H]^+^ = 195.08792 *m*/*z*) and a 2.9-fold increase in theobromine ([M+H]^+^ = 181.07225 *m*/*z*) highlight CSE’s stimulatory effect, which may improve alertness and influence metabolic rate.

The pathway analysis indicated that the metabolites that showed significant differences between baseline and day 7 were those related to glycerophospholipid, caffeine, glycine, serine and threonine, arginine and proline, and linoleic acid metabolism (Figure 7C). These results suggest a reconfiguration of lipid and amino acid metabolism due to CSE supplementation. Additionally, enrichment in xanthines and cholines (key components in neurochemical and membrane dynamics), 6-aminopurines (indicative of nucleotide turnover), and sphingosines (suggestive of changes in lipid signaling molecules) was observed. This indicates broad-spectrum metabolic modulation, affecting both energy and structural molecule pathways. Although less pronounced, amino acids also had a role in the metabolic adaptation observed in this study (Figure 7D).

Overall, the results demonstrate significant metabolic reconfiguration induced by CSE supplementation, affecting multiple pathways related to lipid metabolism, amino acid turnover, and energy homeostasis.

### 3.4. Network Analysis Elucidated Metabolic Pathways Altered by CSE Supplementation

**Figure 8:**
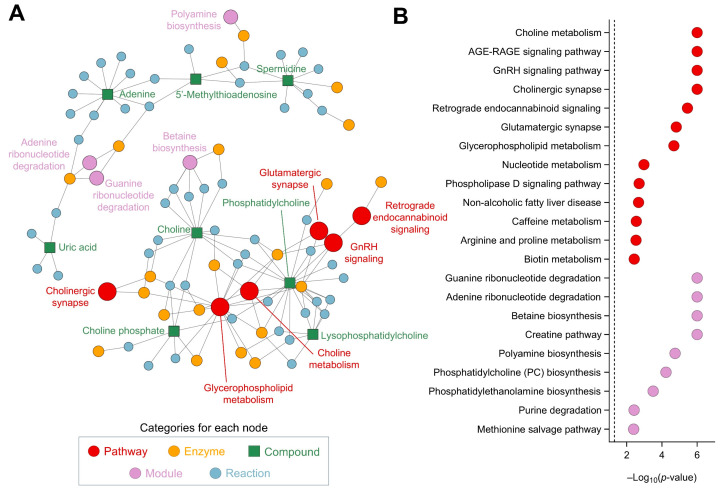
Network and pathway analysis, using FELLA, of the metabolites affected by CSE supplementation, including a network representation of the metabolic pathways, modules, enzymes, and reactions impacted by CSE supplementation (**A**), and a dot plot of the significantly affected pathways and modules, showing their −log10(*p*-value) scores (**B**). Nodes represent different categories: pathways (red, ●), enzymes (yellow, ●), compounds (green, ■), modules (purple, ●), and reactions (blue, ●). The size of the nodes corresponds to the significance of their involvement, with larger nodes indicating higher relevance.

The metabolic analysis conducted highlighted several significant pathways and modules, each contributing to a complex network of biochemical interactions (Figure 8A). The analysis of metabolic pathways revealed notable changes in the cholinergic synapse (*p* = 1.0 × 10^−6^) and glycerophospholipid metabolism (*p* = 2.1 × 10^−5^), crucial for maintaining cellular communication, maintaining neurotransmission, and influencing cognitive functions and muscle control (Figure 8B). The retrograde endocannabinoid signaling (*p* = 3.5 × 10^−6^) and glutamatergic synapse pathway (*p* = 1.5 × 10^−5^) indicate significant changes in neurotransmission processes. Endocannabinoid signaling modulates various physiological processes, including pain sensation, mood, appetite, and memory, while the glutamatergic synapse pathway is involved in excitatory neurotransmission, crucial for synaptic plasticity and cognitive functions. The phospholipase D signaling pathway (*p* = 1.9 × 10^−3^) also showed significant alterations, underscoring disruptions in lipid signaling processes, which are critical in cell growth, differentiation, and immune responses, as they generate phosphatidic acid. Additionally, the nucleotide metabolism pathway (*p* = 1.0 × 10^−3^) and caffeine metabolism pathway (*p* = 2.8 × 10^−3^) reflect energy and purine metabolism shifts, respectively. In terms of metabolic modules, the creatine pathway (*p* = 1.0 × 10^−6^) and betaine biosynthesis pathway (*p* = 1.0 × 10^−6^) are crucial for cellular energy storage and methylation reactions, which are vital for energy production in muscle and brain tissues (Figure 8B). Betaine biosynthesis is important for the methylation of homocysteine to methionine. The methionine salvage pathway (*p* = 4.1 × 10^−3^) and phosphatidylcholine biosynthesis (*p* = 5.9 × 10^−5^) highlight the importance of sulfur amino acid metabolism and phospholipid synthesis. The phosphatidylethanolamine biosynthesis via ethanolamine (*p* = 3.2 × 10^−4^) and phosphatidylethanolamine biosynthesis via phosphatidylserine decarboxylase (*p* = 4.6 × 10^−3^) emphasize significant lipid metabolic shifts. Finally, the purine degradation pathway (*p* = 3.8 × 10^−3^) and adenine ribonucleotide degradation pathway (*p* = 1.0 × 10^−6^) suggest alterations in nucleotide turnover, critical for cellular proliferation and energy metabolism, which are essential for maintaining nucleotide balance and energy homeostasis. The guanine ribonucleotide degradation pathway (*p* = 1.0 × 10^−6^) and polyamine biosynthesis (*p* = 1.8 × 10^−5^) indicate changes in cell growth and differentiation processes.

Overall, the integrated analysis of pathways and modules highlights a broad spectrum of metabolic alterations. These findings underscore the complexity of metabolic regulation and the significant impact of metabolic changes on cellular and systemic functions. This analysis brings forward key insights into the altered biochemical landscape, paving the way for a deeper understanding of metabolic diseases and potential intervention points. The observed changes in the plasma metabolome suggest that CSE has the potential to influence key physiological processes, supporting its use as a nutraceutical with diverse health benefits.

## 4. Discussion

The present study evaluates the metabolic changes induced by CSE supplementation in rats for the first time. Our findings reveal significant modifications in metabolites associated with glycerophospholipid metabolism, amino acid processing, and methylxanthine bioavailability, indicating a multifaceted effect of the CSE on physiological pathways. The distinctive clusters observed for baseline, day 4, and day 7 not only demonstrate the time-dependent metabolic changes caused by CSE, but also highlight the speed with which these changes occurred. Interestingly, as early as day 7, the plasma metabolome had undergone considerable reconfiguration, indicating the significant metabolic effect of a CSE supplementation. Secondly, by analyzing methylxanthines’ bioavailability in rat plasma, we were able to provide insight into CSE’s caffeine and theobromine metabolic rate. The presence of caffeine and theobromine in rats’ plasma confirms the effective absorption and systemic distribution of these methylxanthines after CSE administration. Our analysis identified significant alterations in several key pathways: the cholinergic synapse, glycerophospholipid metabolism, retrograde endocannabinoid signaling, glutamatergic synapse, and phospholipase D signaling. These pathways are vital for cellular communication, neurotransmission, cognitive functions, and muscle control. The observed changes suggest disruptions in lipid signaling, which is crucial for processes such as cell growth and differentiation, immune responses, energy and purine metabolism, membrane structure, protein synthesis, and inflammation regulation. These metabolic changes underline the broad-spectrum influence of CSE on metabolism homeostasis.

Our results indicate that the metabolic profile was not markedly different between the basal time point and day 4 of supplementation, suggesting that a short period of CSE supplementation may not be sufficient to induce marked changes in metabolic pathways. Nonetheless, we observed that caffeine metabolism was detectable in the rat plasma on day 4, demonstrating the bioavailability of methylxanthines (caffeine and theobromine) present in CSE. The presence of caffeine may explain the antioxidant properties and vasodilatation that we have previously shown in vascular tissue with CSE or caffeine supplementation [5]. Caffeine’s mechanism for enhancing vasodilation likely involves inhibiting phosphodiesterase, leading to an increase in cAMP within vascular smooth muscle cells, which promotes relaxation. Additionally, the antioxidant effects of caffeine may derive from its capability to modulate signaling pathways that activate endogenous antioxidant defenses [22]. The presence of caffeine may also contribute to the blood pressure-lowering effects of 2-week CSE supplementation in vivo by upregulating endothelial nitric oxide synthase (e-NOS) and the antioxidant response element nuclear factor (erythroid-derived 2)-like 2 (Nrf2) [16]. It has traditionally been considered that caffeine should be approached with caution in the context of hypertension; however, recent epidemiological data indicate that moderate and habitual consumption of caffeinated coffee does not adversely affect blood pressure and protects against cardiovascular diseases [23,24], which is in agreement with the blood-pressure-lowering effects of CSE supplementation observed in rats.

An increase in glycerophospholipid metabolism was one of the relevant metabolic modifications observed after CSE supplementation, likely reflecting changes in membrane fluidity and signaling that are critical for cardiovascular health and cognitive function. Particularly, the decrease in PC O-20:5 and DHA methyl ester suggests increased utilization of omega-3 fatty acids, altering lipid-mediated signaling and inflammation. These fatty acids, incorporated in cell membranes, are substrates for specialized pro-resolving mediators (SPMs), which inhibit platelets, release NO, and reduce inflammation [25,26]. Omega-3 fatty acids are also essential for neuronal function and cognitive health, contributing to synaptic plasticity and neurotransmission [27,28]. The increase in phosphatidylcholine biosynthesis supports the synthesis of acetylcholine, a key neurotransmitter involved in learning and memory, and has been linked to improved cognitive function [29]. Furthermore, the increase in lysophosphatidylcholines, such as LPC O-13:1, points to dynamic changes in cell membrane compositions that might enhance endothelial function and contribute to improved vascular responses. Plasma lysophosphatidylcholines are negatively correlated with inflammatory markers in patients with myocardial infarction [30], and are also decreased in atherosclerosis and vascular damage [31]. In addition, lysophosphatidylcholines have been shown to influence cognitive function and neurotransmission by acting on G-protein-coupled receptors and modulating synaptic activity, thereby promoting neuronal health [32,33]. The abovementioned metabolic shifts could directly contribute to the observed improvements in cardiovascular function and cognitive health, thereby supporting the potential benefits of CSE supplementation.

Other metabolomic studies evidence that supplementation with polyphenol-rich plants exerts important modifications in glycerophospholipid metabolism, improving obesity-related alterations [34,35]. The changes in sphingolipid metabolism, particularly ceramide levels, indicate a significant impact of CSE on cellular signaling. Ceramides regulate cell membranes, apoptosis, and signal transduction [36]. Their modulation by CSE may enhance cellular resilience to oxidative stress, mitigating inflammation and reducing oxidative damage, as shown in our previous studies in vitro in cell culture models and ex vivo in arteries [4,5]. These effects are particularly relevant in the context of inflammation and oxidative stress illnesses, such as cardiovascular diseases and metabolic disorders, contributing to reducing blood pressure in aged hypertensive animals [16].

The observed decrease in plasmatic amino acids, such as tyrosine and creatine, marks a significant adaptation in nitrogen balance and energy metabolism following CSE supplementation. Reducing tyrosine, a precursor to neurotransmitters like dopamine and norepinephrine, could suggest alterations in catecholamine metabolism [37,38]. Additionally, CSE’s modulation of choline metabolism suggests impacts on neural processes and muscle function. As a key component in acetylcholine synthesis, essential for brain and muscle function, enhanced choline turnover could imply improved cognitive and neural communication due to CSE’s bioactive compounds [39]. Moreover, the decrease in creatine, which is crucial for energy storage and transfer, suggests a shift in energy management strategies. Reduced creatine could indicate increased fatty acid oxidation or enhanced glucose metabolism, enhancing metabolic flexibility [40,41]. Hence, these adjustments, influenced by CSE’s bioactive compounds, may optimize energy usage and neurotransmitter balance to enable individuals to better cope with physiological stressors.

Furthermore, decreased glutathione metabolism post-CSE supplementation may indicate reduced oxidative stress due to CSE’s antioxidant properties. While increased glutathione is typically linked to enhanced antioxidant defense, a decrease might suggest that CSE’s (poly)phenols or methylxanthines mitigate oxidative challenges, reducing reliance on glutathione. This reflects an adaptive optimization of the cellular antioxidant system. CSE intake has also been shown to improve the Nrf2 pathway in cardiovascular tissue and increase GSH in plasma from aged hypertensive rats [16]. The changes in nicotinate and nicotinamide metabolism impact cellular health. Nicotinamide adenine dinucleotide (NAD^+^), a product of this pathway, is vital for energy production and serves as a substrate for DNA repair enzymes [42]. Simultaneously, alterations in 6-aminopurine metabolism, particularly adenine, highlight an effect on purine metabolism. These alterations suggest that CSE supplementation enhances cellular repair and regeneration, helping to maintain cellular integrity under metabolic stress [43]. Additionally, CSE is rich in (poly)phenols with antioxidant and anti-inflammatory properties. Although not directly detected in rat plasma due to the analytical methods employed, their biochemical influence likely contributed to observed metabolic changes. Cocoa shell (poly)phenols can enhance antioxidant defenses, modulate endogenous systems like glutathione [44], regulate gene expression related to metabolism and inflammation [4], and influence lipid and energy metabolism pathways [45].

This research offers noteworthy preliminary findings on the impact of cocoa shell intake on the plasma metabolome in rats. However, there are significant limitations to consider. Firstly, metabolic responses can differ between species, which might lead to inconsistencies when extrapolating results from rats to humans. Secondly, the analysis was primarily focused on plasma metabolome changes, which may ignore potential impacts on other tissues or fluids. Thirdly, the study was conducted exclusively in female rats over a short-term period (7 days), limiting conclusions regarding potential sex-dependent differences or long-term metabolic effects. Future studies should assess male subjects and extended supplementation durations to evaluate these factors. Fourthly, the study used an untargeted metabolomics approach, which, despite allowing an unbiased exploration of the metabolome, might fail to detect certain metabolites that exist in low concentrations or are better suited to a targeted investigation. Additionally, the study only used LC-QTOF analysis in the ESI positive mode, possibly missing data obtainable from other modes or analytical techniques. Considering these limitations and initial results, several ideas for future research emerge. Human-based studies are especially important for expanding our understanding of cocoa shell metabolism and determining its practical health implications. Furthermore, studies should investigate the effects of cocoa shell intake on other physiological systems, such as the gut microbiome, given its role in metabolizing dietary compounds and its influence on overall health. Future research would benefit from using a targeted metabolomics approach for a more precise investigation of specific pathways or molecules of interest altered by the consumption of cocoa shell. Further studies should include longitudinal trials to examine any long-term effects of cocoa shell intake.

Our study provides preliminary insights into the metabolic effects of CSE supplementation, demonstrating significant changes in the plasma metabolome after a 7-day intervention. These changes, mainly affecting glycerophospholipid, amino acid, and fatty acid metabolism, might indicate potential anti-inflammatory and antioxidant activity. The presence of methylxanthines like caffeine and theobromine suggests their contribution to these effects, aligning with their effects on blood pressure regulation, cardiovascular protection, and cognitive function. Thus, our findings support the hypothesis that CSE intake induces metabolic reconfiguration with possible health benefits, offering novel insights into its potential as a bioactive food ingredient or nutraceutical.

## 5. Conclusions

This study demonstrated that CSE supplementation influenced the plasma metabolome of female rats, specifically via modulating lipid metabolism, amino acid pathways, and methylxanthine bioavailability. The metabolic alterations indicate potential functional properties of CSE; however, due to species-specific differences, any extrapolation to humans must be undertaken with caution. Future research should investigate long-term supplementation and sex-specific metabolic differences, as well as conducting targeted mechanistic studies in human subjects to assess the wider effects of CSE consumption. Our findings emphasize the practical implications of including CSE, a sustainable cocoa by-product, in dietary interventions targeted at improving metabolic health. This study not only provides new insights into the biological activity of the CSE, but also demonstrates the application of food by-products in promoting health and sustainability.

## Figures and Tables

**Figure 1:**
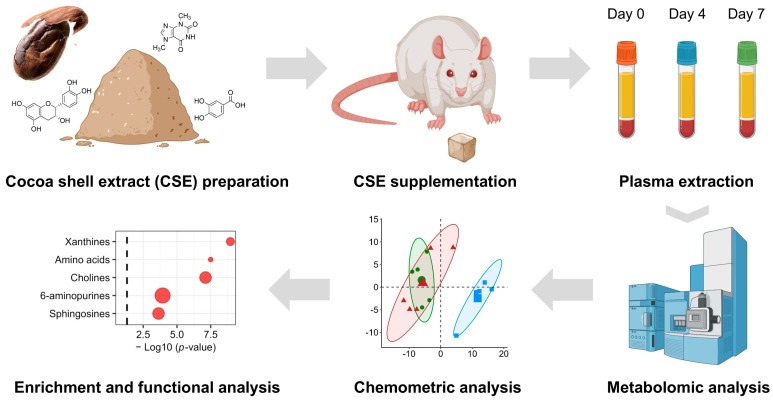
A flow chart of the experimental design. This diagram outlines the steps involved in the experimental procedure for CSE supplementation analysis. Starting with the preparation of the cocoa shell extract (CSE), the chart depicts the subsequent phases, including the administration of CSE to the female rats, the collection of biological samples at defined time points, and the metabolomic analysis. The latter involves mass spectrometry-based metabolite identification, data processing, and subsequent bioinformatics and chemometric analysis, including pathway- and network-based enrichment analyses.

**Table 1:** Comprehensive metabolomic profile of cocoa shell extract (CSE), showing identified metabolites, their chemical families, retention times (R_t_), molecular formulas, adducts, and mass-to-charge ratios (*m*/*z*), along with observed errors and relative abundances.

Metabolite Name	Chemical Family	R_t_ (min)	Formula	Adduct	Observed *m*/*z*	Calculated *m*/*z*	Error (ppm)	Cocoa Shell (Count × 10^4^)
Caffeine	Xanthines	3.073	C_8_H_10_N_4_O_2_	[M+H]^+^	195.08735	195.08771	−1.8	181.8716
Nicotinoylglycine	Amino acids	2.392	C_8_H_8_N_2_O_3_	[2M+H]^+^	181.06001	181.06050	−2.7	176.9846
Choline cation	Cholines	0.569	C_5_H_14_NO	[Cat]^+^	104.10646	104.10700	−5.2	109.5438
Betaine	Amino acids	0.589	C_5_H_12_NO_2_^+^	[M+H]^+^	118.08614	118.08570	3.7	68.2777
Phenylalanine	Amino acids	3.870	C_9_H_11_NO_2_	[M+H]^+^	166.08574	166.08600	−1.6	62.9024
L-Isoleucine	Amino acids	1.323	C_6_H_13_NO_2_	[M+H]^+^	132.10197	132.10190	0.5	59.1781
DL-Norvaline	Amino acids	0.648	C_5_H_11_NO_2_	[M+H]^+^	118.08676	118.08677	−0.1	49.8276
(3-nitrophenyl)methanol	Benzyl alcohols	2.983	C_7_H_7_NO_3_	[M+H]^+^	154.05022	154.04990	2.1	28.7702
*N*-(2-Pyridylmethyl)piperazine-1-ethylamine	Piperazines	0.589	C_12_H_20_N_4_	[M+H]^+^	221.17647	221.17610	1.7	28.0571
DL-Norleucine	Amino acids	2.209	C_6_H_13_NO_2_	[M+H]^+^	132.10193	132.10193	0.0	21.5649
2-Hydroxyphenethylamine	Phenethylamine	2.773	C_8_H_11_NO	[M+H]^+^	120.08086	120.08080	0.5	18.2369
*N*-Acetyl-L-glutamic acid	Amino acids	1.148	C_7_H_11_NO_5_	[M+H]^+^	172.06044	172.06039	0.3	18.2252
3,4-Cyclopropylglutamate	Amino acids	1.043	C_6_H_9_NO_4_	[M+H]^+^	140.03476	140.03529	−3.8	15.7238
Asperglaucide	Dipeptides	7.996	C_27_H_28_N_2_O_4_	[M+Na]^+^	467.19397	467.19400	−0.1	12.9412
Stachydrine	Pyrrolidine alkaloids	0.708	C_7_H_13_NO_2_	[M+H]^+^	144.10246	144.10190	3.9	11.8364
Tris(2,4-di-tert-butylphenyl)phosphate	Organophosphorus compounds	13.926	C_42_H_63_O_4_P	[M+H]^+^	663.45374	663.45367	0.1	10.8888
5-oxo-D-Proline	Amino acids	0.990	C_5_H_7_NO_3_	[M+H]^+^	130.04996	130.04997	−0.1	10.7441
7-Methylxanthine	Xanthines	2.075	C_6_H_6_N_4_O_2_	[M+H]^+^	167.05612	167.05635	−1.4	10.5532
L-Proline	Amino acids	7.990	C_5_H_9_NO_2_	[M+H]^+^	117.06997	117.07000	−0.3	9.5794
1-Aminocyclohexanecarboxylic acid	Amino acids	1.814	C_7_H_13_NO_2_	[M+H]^+^	144.10184	144.10190	−0.4	9.0652
Val-Val	Dipeptides	2.529	C_10_H_20_N_2_O_3_	[M+H]^+^	217.15448	217.15469	−1.0	8.2541
Ugandenial A	Sesquiterpenes	1.911	C_15_H_22_O_4_	[M+H]^+^	289.13965	289.14102	−4.7	8.1922
L-Tyrosine	Amino acids	3.966	C_9_H_11_NO_3_	[M+H]^+^	182.08141	182.08141	0.0	7.7061
Koninginin E	Benzopyrans	1.262	C_16_H_26_O_5_	[M+H]^+^	321.16577	321.16571	0.2	7.3375
D-Pipecolinic acid	Carboxylic acid	3.121	C_6_H_11_NO_2_	[M+H]^+^	259.16425	259.16519	−3.6	6.0300
Leu-Val	Dipeptides	2.622	C_11_H_22_N_2_O_3_	[M+H]^+^	231.17027	231.17030	−0.1	6.0071
5-phenyllevulinic acid	Carboxylic acids	2.864	C_11_H_12_O_3_	[M+K]^+^	210.11296	210.11301	−0.2	5.8234
Leu-Ile	Dipeptides	3.068	C_12_H_24_N_2_O_3_	[M+H]^+^	245.18800	245.18600	8.2	5.7594
(Z)-5,8,11-trihydroxyoctadec-9-enoic acid	Fatty acids	6.224	C_18_H_34_O_5_	[M+NH_4_]^+^	348.27463	348.27341	3.5	5.5588
Piperidine	Piperidines	1.310	C_5_H_11_N	[M+H]^+^	86.09669	86.09694	−2.9	5.5261
Pro-Val	Dipeptides	2.062	C_10_H_18_N_2_O_3_	[M+H]^+^	215.13892	215.13901	−0.4	5.4371
*N*-Acetylproline	Amino acids	2.238	C_7_H_11_NO_3_	[2M+K]^+^	158.08084	158.08119	−2.2	5.4242
1-Palmitoyl-2-oleoylphosphatidylserine	Phospholipids	12.570	C_40_H_76_NO_10_P	[M+Na]^+^	780.55267	780.55249	0.2	5.2930
L-Pipecolic acid	Piperidines	2.308	C_6_H_11_NO_2_	[M+H]^+^	112.07574	112.07570	0.4	5.1864
6-Hydroxynicotinic acid	Pyridine carboxylic acids	1.169	C_6_H_5_NO_3_	[M+H]^+^	140.03412	140.03419	−0.5	5.1694
5-Hydroxy-2-(hydroxymethyl)pyridine	Piridines	0.749	C_6_H_7_NO_2_	[M+H]^+^	126.05507	126.05500	0.6	5.0079
*trans*-2-dodecenedioic acid	Fatty acids	3.313	C_12_H_20_O_4_	[M+Na]^+^	251.12547	251.12540	0.3	4.7774
4-Guanidinobutyric acid	Guanidines	2.388	C_5_H_11_N_3_O_2_	[M+NH_4_]^+^	434.24939	434.24811	2.9	4.5778
Nicotinic acid	Pyridine carboxylic acids	0.897	C_6_H_5_NO_2_	[M+H]^+^	124.03944	124.03943	0.1	4.4967
Ile-Phe	Dipeptides	3.424	C_15_H_22_N_2_O_3_	[M+H]^+^	279.17035	279.17029	0.2	4.3652
L-Tryptophan	Amino acids	2.567	C_11_H_12_N_2_O_2_	[M+H]^+^	205.09715	205.09715	0.0	4.3597
Ser-Tyr-Lys	Tripeptides	2.187	C_18_H_28_N_4_O_6_	[M+NH_4_]^+^	199.10767	199.10770	−0.2	4.2192
PE 17:0/22:6	Phospholipids	12.401	C_44_H_75_O_8_P	[M+H]^+^	780.55450	780.55383	0.9	4.1227
Procyanidin B2	(Poly)phenols	2.794	C_30_H_26_O_12_	[M+H]^+^	579.14984	579.14972	0.2	4.0849
(+)-Catechin	(Poly)phenols	3.273	C_15_H_14_O_6_	[M+H]^+^	291.08630	291.08630	0.0	4.0531
Aurantiamide	Dipeptides	7.012	C_25_H_26_N_2_O_3_	[M+H]^+^	403.20200	403.20111	2.2	4.0333
1,3,7-Trimethyluric acid	Xanthines	2.754	C_8_H_10_N_4_O_3_	[M+H]^+^	211.08286	211.08299	−0.6	3.7675
4-Methylquinolin-2-ol	Quinolines	0.539	C_10_H_9_NO	[M+K]^+^	319.14383	319.14410	−0.8	3.7625
Gentiobiose	Disaccharide	0.595	C_12_H_22_O_11_	[M+K]^+^	381.07919	381.07938	−0.5	3.7047
MGDG O-9:0/22:6	Galactolipids	13.582	C_40_H_66_O_9_	[M+H]^+^	708.50733	708.50452	4.0	3.7007
Phenylethylamine	Phenylethylamines	2.311	C_8_H_11_N	[M+H−NH_3_]^+^	105.06991	105.06990	0.1	3.6676
2-Aminophenol	Aminophenols	0.961	C_6_H_7_NO	[M+H]^+^	110.06028	110.06020	0.7	3.6554
MGDG 2:0/6:0	Galactolipids	4.118	C_17_H_30_O_10_	[M+NH_4_]^+^	412.21765	412.21771	−0.1	3.6006
Pyrrole-2-carboxylic acid	Pyrrole carboxylic acids	2.984	C_5_H_5_NO_2_	[M+H]^+^	112.03905	112.03930	−2.2	3.5997
Dianthoside	Triterpenes	3.004	C_12_H_16_O_8_	[2M+Na]^+^	289.09192	289.09201	−0.3	3.5732
L-Leucine	Amino acids	1.956	C_6_H_13_NO_2_	[M+H]^+^	132.10172	132.10172	0.0	3.5372
Trigonelline	Alkaloids	0.611	C_7_H_7_NO_2_	[M+H]^+^	138.05518	138.05495	1.7	3.5338
3,7-Dihydroxyflavone	(Poly)phenols	2.421	C_15_H_10_O_4_	[2M+H]^+^	255.1194	255.11984	−1.7	3.4200
cAMP	Ribonucleotides	1.967	[C_10_H_11_N_5_O_6_P]^−^	[M+Na]^+^	268.10422	268.10410	0.4	3.3707
Ferulamide	(Poly)phenols	3.196	C_10_H_11_NO_3_	[M+H]^+^	194.08067	194.08099	−1.6	3.3350
Pro-Ile	Dipeptides	1.132	C_11_H_20_N_2_O_3_	[M+H]^+^	229.15486	229.15469	0.7	3.3262
PC 8:0/30:7	Phospholipids	11.867	C_46_H_78_NO_8_P	[M+Na]^+^	804.55298	804.55377	−1.0	3.3210
Pantothenic acid	Carboxylic acids	2.273	C_9_H_17_NO_5_	[M+H]^+^	220.11768	220.11760	0.4	3.2063
1,2-Dipalmitoyl-sn-glycero-3-phospho-(1′-rac-glycerol)	Phospholipids	13.668	C_38_H_75_O_10_P	[M+H]^+^	721.50586	721.50250	4.7	3.1628
5-Aminovaleric acid	Amino acids	2.134	C_5_H_11_NO_2_	[M+H]^+^	100.07556	100.07570	−1.4	3.1572
MGDG O-8:0/12:0	Galactolipids	9.514	C_29_H_56_O_9_	[M+H]^+^	566.42761	566.42627	2.4	3.0768
Val-Phe	Dipeptides	2.987	C_14_H_20_N_2_O_3_	[M+H]^+^	265.15451	265.15469	−0.7	2.9783
Theobromine	Xanthines	2.589	C_7_H_8_N_4_O_2_	[M+H]^+^	181.07198	181.07201	−0.2	2.9773
4-Acetamidobutanoic acid	Amino fatty acids	1.912	C_6_H_11_NO_3_	[M+H]^+^	146.0809	146.08118	−1.9	2.9351
3-Hydroxy-2-methylpyridine	Pyridine alkaloids	0.738	C_6_H_7_NO	[M+H]^+^	110.06017	110.06000	1.5	2.8867
1,2-Diamino-2-methylpropane	Alkylamines	2.667	C_4_H_12_N_2_	[M+H]^+^	72.08076	72.08080	−0.6	2.8804
2,6-Dihydroxypyridine	Pyridine alkaloids	1.010	C_5_H_5_NO_2_	[M+H]^+^	112.03943	112.03933	0.9	2.8761
4-Hydroxyquinoline	Quinolines	2.731	C_9_H_7_NO	[M+H]^+^	146.06039	146.06090	−3.5	2.8048
Citric acid	TCA acid	0.965	C_6_H_8_O_7_	[M+H]^+^	193.03419	193.03430	−0.6	2.7578
Glu-Ile-Arg	Tripeptides	2.436	C_17_H_30_N_6_O_5_	[2M+H]^+^	399.23471	399.23499	−0.7	2.7442
2-Aminobenzoic acid	Carboxylic acids	2.843	C_7_H_7_NO_2_	[M+H]^+^	138.05518	138.05499	1.4	2.7030
Guanosine	Purine Nucleosides	1.999	C_10_H_13_N_5_O_5_	[M+K]^+^	284.09915	284.09906	0.3	2.6956
4-Hydroxyphenethylamine	Phenethylamines	1.425	C_8_H_11_NO	[M+H]^+^	138.09138	138.09129	0.7	2.6598
(−)-Epicatechin	(Poly)phenols	2.632	C_15_H_14_O_6_	[M+H]^+^	291.08655	291.08633	0.8	2.6549
3,4,5-Trihydroxyphenethylamine	Phenethylamines	1.006	C_8_H_11_NO_3_	[M+H]^+^	168.06577	168.06660	−4.9	2.6351
Sorbicillin	Phenols	1.522	C_14_H_16_O_3_	[M+H]^+^	231.10338	231.10271	2.9	2.6254
Ala-Ile	Dipeptides	2.237	C_9_H_18_N_2_O_3_	[M+H]^+^	203.1384	203.13850	−0.5	2.5838
3-Hydroxypicolinic acid	Pyridines	2.983	C_6_H_5_NO_3_	[M+H]^+^	122.02402	122.02370	2.6	2.5708
2-(2-Ethylbutanoylamino)-4,5-dimethoxybenzoic acid	Carboxylic acids	3.164	C_15_H_21_NO_5_	[M+H]^+^	250.14453	250.14490	−1.5	2.5577
*p*-Carboxymethylphenylalanine	Amino acids	2.215	C_11_H_13_NO_4_	[M+H]^+^	224.09134	224.09171	−1.7	2.4812
(9Z,12E)-15,16-dihydroxyoctadeca-9,12-dienoic acid	Fatty acids	6.950	C_18_H_32_O_4_	[M+H]^+^	313.2373	313.23734	−0.1	2.4777
*N*-Isovaleroylglycine	Amino acids	0.738	C_7_H_13_NO_3_	[M+H]^+^	160.09671	160.09680	−0.6	2.4453
(+)-Cathinone	Alkaloids	4.316	C_9_H_11_NO	[M+H]^+^	299.17529	299.17542	−0.4	2.4319
*N*-Acetyl-β-D-mannosamine	Amino sugar	1.101	C_8_H_15_NO_6_	[M+H−C_2_H_8_O_4_]^+^	126.05508	126.05500	0.6	2.4197
4-Aminophenol	Aminophenols	2.388	C_6_H_7_NO	[M+H]^+^	110.05943	110.05890	4.8	2.4185
Kynurenic acid	Xanthurenates	2.824	C_10_H_7_NO_3_	[M+H]^+^	190.04955	190.04950	0.3	2.3696
Pro-Trp	Dipeptides	3.067	C_16_H_19_N_3_O_3_	[M+H]^+^	302.15033	302.14990	1.4	2.3584
Anabasamine	Alkaloids	2.069	C_16_H_19_N_3_	[M+Na]^+^	254.16515	254.16518	−0.1	2.3187
MGDG 2:0/3:0	Galactolipids	2.606	C_14_H_24_O_10_	[M+NH_4_]^+^	370.17099	370.17078	0.6	2.3122
Harmine	Alkaloids	2.428	C_13_H_12_N_2_O	[M+H]^+^	213.11624	213.11636	−0.6	2.1951
*N*-*cis*-*p*-Coumaroyltyrosine	(Poly)phenols	4.062	C_18_H_17_NO_5_	[M+H]^+^	328.11798	328.11795	0.1	2.1564
Guanine	Purine	2.000	C_5_H_5_N_5_O	[M+Na]^+^	152.05716	152.05721	−0.3	2.1498
SM (34:1;O2)	Phospholipids	13.742	C_37_H_63_N_2_O_6_P	[M+H]^+^	685.43457	685.43158	4.4	2.1476
Kavain	Pyrans	1.231	C_14_H_14_O_3_	[M+H]^+^	231.10153	231.10156	−0.1	2.0343
Jaeschkeanadiol	Sesquiterpenes	8.098	C_15_H_26_O_2_	[M+H]^+^	256.22699	256.22708	−0.4	1.9400
4,6-Dioxoheptanoic acid	Fatty acids	2.754	C_7_H_10_O_4_	[M+H]^+^	141.05466	141.05460	0.4	1.9085
Sclareol	Diterpenoids	11.403	C_20_H_36_O_2_	[M+H]^+^	326.3049	326.30533	−1.3	1.8766
Cyclo(Leu-Pro)	Dipeptides	3.570	C_11_H_18_N_2_O_2_	[M+H]^+^	211.14395	211.14391	0.2	1.8738
Tyr-Gly-Gly-Phe-Leu	Peptides	7.012	C_28_H_37_N_5_O_7_	[M+Na]^+^	425.18323	425.18188	3.2	1.8470
Methacholine	Cholines	0.852	C_8_H_18_NO_2_	[M+H]^+^	160.13336	160.13319	1.1	1.8445
Hypoxanthine	Xanthines	1.030	C_5_H_4_N_4_O	[M+H]^+^	137.04565	137.04570	−0.4	1.8251
Val-Tyr	Dipeptides	2.287	C_14_H_20_N_2_O_4_	[M+H]^+^	281.14932	281.14960	−1.0	1.8210
Leu-Gly	Dipeptides	1.991	C_8_H_16_N_2_O_3_	[M+H]^+^	189.12323	189.12340	−0.9	1.8165
*N*-Carboxyethyl-γ-aminobutyric acid	Amino acids	0.807	C_7_H_13_NO_4_	[M+H]^+^	176.09164	176.09171	−0.4	1.8102
9-Oxo-10E,12Z-octadecadienoic acid	Fatty acids	6.239	C_18_H_30_O_3_	[M+H]^+^	295.22675	295.22681	−0.2	1.8013
Oxazole-4-carboxylic acid	Carboxyl acids	0.472	C_4_H_3_NO_3_	[M+H]^+^	112.00468	112.00400	6.1	1.8009
L-Saccharopine	Amino acids	0.985	C_11_H_20_N_2_O_6_	[M+H]^+^	275.12485	275.12491	−0.2	1.7431
Phe-Gly	Dipeptides	2.294	C_11_H_14_N_2_O_3_	[M+NH_4_]^+^	223.10759	223.10770	−0.5	1.7342
L-Carnitine	Amino acids	0.603	C_7_H_15_NO_3_	[M+H]^+^	162.11243	162.11000	15.0	1.6820
Ala-Val	Dipeptides	0.950	C_8_H_16_N_2_O_3_	[M+H]^+^	189.12366	189.12340	1.4	1.6673
Arg-Gln	Dipeptides	1.025	C_11_H_22_N_6_O_4_	[M+H]^+^	286.15097	286.15100	−0.1	1.6572
Phe-Pro	Dipeptides	3.055	C_14_H_18_N_2_O_3_	[M+H]^+^	263.13907	263.13901	0.2	1.6562
Phytosphingosine	Phytosphingosines	7.893	C_18_H_39_NO_3_	[M+H]^+^	318.30017	318.30020	−0.1	1.6243
5-(Carbamoylamino)pentanoic acid	Amino acids	2.226	C_6_H_12_N_2_O_3_	[M+H]^+^	100.07606	100.07570	3.6	1.5755
(Z)-3-Hexenylvicianoside	O-acyl carbohydrate	4.237	C_17_H_30_O_10_	[M+H]^+^	412.21793	412.21799	−0.1	1.5699
4-Methylamino-4-pyridin-3-ylbutanoic acid	Carboxyl acids	2.563	C_10_H_14_N_2_O_2_	[M+H]^+^	195.11305	195.11279	1.3	1.5554
2-Pyrrol-1-ylbenzoic acid	Carboxyl acids	2.566	C_11_H_9_NO_2_	[M+H]^+^	188.07144	188.07060	4.5	1.5549
3-[(2S,5S)-5-(2-methylpropyl)-3,6-dioxopiperazin-2-yl]propanoic acid	Carboxyl acids	2.616	C_11_H_18_N_2_O_4_	[M+NH_4_]^+^	243.13394	243.13390	0.2	1.5236
Ala-Nle	Dipeptides	2.097	C_9_H_18_N_2_O_3_	[M+NH_4_]^+^	203.13895	203.13901	−0.3	1.5089
*N*-Acetylleucine	Amino acids	3.411	C_8_H_15_NO_3_	[M+H]^+^	174.11224	174.11230	−0.3	1.4734
PC(16:0–18:0)	Phospholipids	13.303	C_42_H_80_NO_8_P	[M+H]^+^	780.55164	780.55139	0.3	1.4565
Ile-Ala	Dipeptides	1.954	C_9_H_18_N_2_O_3_	[2M+H]^+^	203.13898	203.13901	−0.1	1.4533
4-Oxobedfordiaic Acid	Sesquiterpenes	2.107	C_15_H_22_O_3_	[M+H]^+^	273.14401	273.14600	−7.3	1.4424
*N*-Acetyltyramine	Biogenic monoamines	3.050	C_10_H_13_NO_2_	[M+H]^+^	180.10194	180.10190	0.2	1.4384
3-Amino-L-tyrosine	Amino acids	2.988	C_9_H_12_N_2_O_3_	[M+H]^+^	393.17627	393.17691	−1.6	1.4333
L-Cysteine	Amino acids	0.464	C_3_H_7_NO_2_S	[M+H]^+^	105.00085	105.00050	3.3	1.4317
PyroGlu-Val	Dipeptides	2.578	C_10_H_16_N_2_O_4_	[M+K]^+^	229.11827	229.11830	−0.1	1.4314
γ-Glu-Glu	Dipeptides	2.407	C_10_H_16_N_2_O_7_	[M+K]^+^	260.07739	260.07651	3.4	1.4300
γ-Glu-Phe	Dipeptides	1.883	C_14_H_16_N_2_O_4_	[M+K]^+^	277.11807	277.11829	−0.8	1.4289
*N*-Acetylphenylalanine	Amino acids	3.880	C_11_H_13_NO_3_	[M+H]^+^	208.0966	208.09660	0.0	1.4199
6-Oxooctadecanoic acid	Medium and short-chain keto acids	10.702	C_18_H_34_O_3_	[M+H]^+^	316.28479	316.28461	0.6	1.3920
Ile-Ser	Dipeptides	2.157	C_9_H_18_N_2_O_4_	[M+Na]^+^	217.11902	217.11940	−1.8	1.3857
Leu-Glu	Dipeptides	1.913	C_11_H_20_N_2_O_5_	[M+Na]^+^	261.14471	261.14450	0.8	1.3560
L-Homoserine	Amino acids	2.413	C_4_H_9_NO_3_	[2M+K]^+^	237.10887	237.10921	−1.4	1.3545
*N*-(*tert*-Butoxycarbonyl)glycine	Amino acids	2.388	C_7_H_13_NO_4_	[M+H]^+^	176.09175	176.09171	0.2	1.3386
*N-*(2-Hydroxyethyl)iminodiacetic acid	Dicarboxilic acids	0.476	C_6_H_11_NO_5_	[M+H]^+^	160.06079	160.06039	2.5	1.3136
3-Methyladipic acid	Dicarboxilic acids	2.382	C_7_H_12_O_4_	[M+H]^+^	183.06261	183.06281	−1.1	1.3110
N-Acetyl-DL-valine	Amino acids	2.692	C_7_H_13_NO_3_	[M+H]^+^	160.09645	160.09680	−2.2	1.3057
Tazettine	Alkaloids	2.282	C_18_H_21_NO_5_	[M+H]^+^	332.15002	332.14926	2.3	1.3057
Phe-Ile	Dipeptides	2.282	C_15_H_22_N_2_O_3_	[2M+Na]^+^	279.17029	279.17029	0.0	1.2998
Ser-Leu	Dipeptides	0.819	C_9_H_18_N_2_O_4_	[M+H]^+^	219.134	219.13390	0.5	1.2740
Leu-Val-Pro	Tripeptides	3.077	C_16_H_29_N_3_O_4_	[M+H]^+^	328.22336	328.22311	0.8	1.2653
L-Homocitrulline	Amino acids	1.921	C_7_H_15_N_3_O_3_	[M+H]^+^	212.10071	212.10060	0.5	1.2619
Ethyl L-leucinate	Amino acids	2.338	C_7_H_15_NO_2_	[M+H]^+^	160.13255	160.13300	−2.8	1.2483
Linoleoyl ethanolamide	*N*-acylethanolamines	10.574	C_20_H_37_NO_2_	[M+H]^+^	324.28983	324.28970	0.4	1.2458
Methyl 3,4,5-trihydroxycyclohexene-1-carboxylate	Carbonyls	1.753	C_8_H_12_O_5_	[M+H]^+^	230.10199	230.10201	−0.1	1.2432
PC (16:0/22:5)	Phospholipids	11.864	C_46_H_82_NO_8_P	[M+H]^+^	830.56665	830.56702	−0.4	1.2241
Ala-Phe	Dipeptides	2.523	C_12_H_16_N_2_O_3_	[M+H]^+^	237.12341	237.12340	0.0	1.2230
*trans*-Cinnamic acid	(Poly)phenols	2.139	C_9_H_8_O_2_	[M+H]^+^	149.05951	149.05971	−1.3	1.2199
*O*-Benzyl-L-serine	Amino acids	1.990	C_10_H_13_NO_3_	[M+H]^+^	196.09682	196.09680	0.1	1.2078
5-Methoxytryptophan	Amino acids	2.198	C_12_H_14_N_2_O_3_	[M+H]^+^	235.1078	235.10770	0.4	1.1960
His-Thr-Lys	Tripeptides	4.882	C_16_H_28_N_6_O_5_	[M+Na]^+^	367.20892	367.20880	0.3	1.1949
Scalusamide A	Pyrrolidines	1.923	C_16_H_27_NO_3_	[M+K]^+^	304.18692	304.18799	−3.5	1.1937
Lys-Asn	Dipeptides	2.072	C_10_H_20_N_4_O_4_	[M+H]^+^	283.13898	283.13770	4.5	1.1887
2-Pyridinecarboxaldehyde	Pyridine	0.602	C_6_H_5_NO	[M+H]^+^	108.04469	108.04440	2.7	1.1767
4-(Butylamino)benzoic acid	Carbocyclic acids	7.993	C_11_H_15_NO_2_	[M+H]^+^	194.11751	194.11760	−0.5	1.1756
Glu-Pro	Dipeptides	0.941	C_10_H_14_N_2_O_4_	[M+H]^+^	227.10255	227.10260	−0.2	1.1669
His-Pro	Dipeptides	2.329	C_11_H_16_N_4_O_3_	[M+H]^+^	253.12955	253.12950	0.2	1.1635
Desmotroposantonin	Terpenes	5.085	C_15_H_18_O_3_	[M+H]^+^	247.13298	247.13300	−0.1	1.1550
γ-Glu-Val	Dipeptides	0.632	C_10_H_18_N_2_O_5_	[M+H]^+^	247.20084	247.20000	3.4	1.1543
MGDG O-8:0/2:0	Galactolipids	2.453	C_19_H_36_O_9_	[2M+H]^+^	431.22403	431.22516	−2.6	1.1503
L-Propionylcarnitine	Amino acids	2.100	C_10_H_19_NO_4_	[M+H]^+^	240.11928	240.12061	−5.5	1.1452
*N*6-Succinyladenosine	Purine Nucleosides	2.301	C_14_H_17_N_5_O_8_	[M+K]^+^	384.11508	384.11499	0.2	1.1387
*N*-ɑ-Acetyl-L-arginine	Amino acids	0.873	C_8_H_16_N_4_O_3_	[2M+H]^+^	217.12956	217.12950	0.3	1.1374
L-Citrulline	Amino acids	0.543	C_6_H_13_N_3_O_3_	[M+Na]^+^	159.07658	159.07640	1.1	1.1237
Phe-Ala-Lys	Tripeptides	2.583	C_18_H_28_N_4_O_4_	[M+H]^+^	183.11249	183.11279	−1.6	1.1080
*N*5-(1-Iminoethyl)-L-ornithine	Amino acids	1.895	C_7_H_15_N_3_O_2_	[M+H]^+^	174.12317	174.12370	−3.0	1.0954
7-Keto-8-aminopelargonic acid	Amino acids	3.566	C_9_H_17_NO_3_	[M+NH_4_]^+^	170.11769	170.11760	0.5	1.0777
4-Methyl-2-oxovaleric acid	Medium and short-chain keto acids	2.632	C_6_H_10_O_3_	[M+H]^+^	113.05968	113.05970	−0.2	1.0733
Val-Trp	Dipeptides	3.233	C_16_H_21_N_3_O_3_	[M+Na]^+^	304.16547	304.16559	−0.4	1.0630
Glu-Ala-Lys	Tripeptides	1.111	C_14_H_24_N_4_O_5_	[M+H]^+^	329.18179	329.18188	−0.3	1.0484
O-Phospho-L-threonine	Amino acids	0.575	C_4_H_10_NO_6_P	[M+K]^+^	222.01401	222.01379	1.0	1.0336
*N*-Acetylmannosamine	Amino sugar	2.389	C_8_H_15_NO_6_	[2M+NH_4_]^+^	222.09795	222.09810	−0.7	1.0325
2′-Deoxyuridine	Pyrimidine	1.111	C_9_H_12_N_2_O_5_	[M+H]^+^	211.07178	211.07130	2.3	1.0163
Gly-Ile	Dipeptides	0.811	C_8_H_16_N_2_O_3_	[M+H]^+^	189.12372	189.12350	1.2	1.0063
Leu-Leu	Dipeptides	2.845	C_12_H_24_N_2_O_3_	[M+H]^+^	245.186	245.18600	0.0	0.6975

SM: Sphingomyelin; PC: Phosphatidylcholine; MGDG: Monogalactosyldiacylglycerol; PE: Phosphatidylethanolamine; cAMP: Cyclic adenosine monophosphate.

**Table 2:** Differentially abundant metabolites in plasma following CSE Supplementation. This table presents the metabolites identified through untargeted metabolomic analysis, highlighting their retention time (*R_t_*), molecular formula, adduct type, observed and calculated mass-to-charge ratios (*m*/*z*), mass error (in ppm), relative intensity (counts × 10^4^), fold change (FC) between basal and day 7 measurements, *p*-value, and false discovery rate (FDR). The data emphasize significant metabolic changes over the CSE supplementation period, providing insights into the compounds that exhibit differential abundance.

Metabolite Name	*R_t_* (min)	Formula	Adduct	Observed *m*/*z*	Calculated *m*/*z*	Error (ppm)	Relative Intensity (Counts × 10^4^)		FC	*p*-Value	FDR
							**Basal**	**Day 7**			
1-(4-methylsulfanylphenyl)-3-phenylurea	8.378	C_14_H_14_N_2_OS	[M+Na]^+^	517.17255	517.17267	−0.2	0.07 (0.00)	1.86 (0.10)	21.5	0.005	0.017
LPC O-13:1	10.451	C_21_H_44_NO_6_P	[M+H]^+^	438.29791	438.29791	0.0	0.17 (0.00)	1.36 (0.23)	7.5	0.005	0.017
1-(2-Hydroxyethyl)-2,2,6,6-tetramethyl-4-piperidinol	1.224	C_11_H_23_NO_2_	[M+H]^+^	202.18022	202.18021	0.0	0.23 (0.00)	1.76 (0.30)	6.9	0.005	0.017
1,2,3,4-Tetrahydro-b-carboline	3.460	C_11_H_12_N_2_	[M+H]^+^	173.10725	173.10730	−0.3	0.22 (0.00)	1.34 (0.32)	6.0	0.005	0.017
PC (18:0/22:6)	12.403	C_48_H_84_NO_8_P	[M+H]^+^	834.60095	834.60071	0.3	0.20 (0.00)	0.70 (0.19)	5.5	0.005	0.017
13-hydroperoxy-1-piperidin-1-ylicosa-2,4,14-trien-1-one	12.591	C_25_H_43_NO_3_	[M+Na]^+^	406.32959	406.33099	−3.4	1.03 (0.00)	5.31 (0.81)	4.6	0.005	0.017
D-erythro-*N*-stearoylsphingosine	8.203	C_18_H_37_NO_2_	[M+H]^+^	300.28961	300.28970	−0.3	0.40 (0.00)	1.16 (0.50)	4.1	0.005	0.017
DGGA 13:0/27:0	5.913	C_49_H_92_O_11_	[M+H]^+^	874.69995	874.69781	2.4	0.28 (0.00)	0.88 (0.12)	3.8	0.005	0.017
Glucose	2.619	C_6_H_12_O_6_	[M+K]^+^	181.07214	181.07204	0.6	0.32 (0.00)	1.13 (0.15)	3.8	0.005	0.017
Docosahexaenoic acid methyl ester	12.889	C_23_H_34_O_2_	[M+H]^+^	343.26376	343.26321	1.6	0.32 (0.00)	1.19 (0.06)	3.5	0.005	0.017
NAGlySer 26:7/17:2	11.875	C_48_H_74_N_2_O_7_	[M+H]^+^	808.58398	808.58344	0.7	0.74 (0.00)	2.13 (1.03)	3.0	0.019	0.050
Theobromine	2.391	C_7_H_8_N_4_O_2_	[M+H]^+^	181.07225	181.07204	1.2	8.12 (0.00)	24.5 (2.30)	2.9	0.005	0.017
*N*-Isovaleroylglycine	2.376	C_7_H_13_NO_3_	[M+H]^+^	182.07898	182.07880	1.0	0.68 (0.17)	1.98 (0.17)	2.8	0.005	0.017
Tripropylene glycol	3.240	C_9_H_20_O_4_	[M+H]^+^	193.14313	193.14340	−1.4	0.52 (1.42)	2.86 (0.75)	2.7	0.016	0.045
Caffeine	3.064	C_8_H_10_N_4_O_2_	[M+H]^+^	195.08792	195.08771	1.1	4.91 (0.00)	12.0 (0.55)	2.5	0.005	0.017
Choline cation	0.569	C_5_H_14_NO	[Cat]^+^	104.10635	104.10700	−6.2	11.0 (0.60)	17.4 (2.05)	1.5	0.016	0.045
2-Methylisoquinolin-2-ium cation	2.131	C_10_H_10_N	[Cat−C_2_H_3_N]^+^	103.05445	103.05420	2.4	3.20 (0.25)	2.90 (0.05)	−1.1	0.016	0.045
Cytidine	0.981	C_9_H_13_N_3_O_5_	[M+H-C_5_H_8_O_4_]^+^	112.05074	112.05050	2.1	11.8 (0.15)	10.8 (0.35)	−1.1	0.009	0.027
Tyrosine	1.379	C_9_H_11_NO_3_	[M+H]^+^	182.08156	182.08141	0.8	9.29 (0.30)	6.12 (0.42)	−1.4	0.016	0.045
Asp-Lys	0.599	C_10_H_19_N_3_O_5_	[M+H]^+^	132.07436	132.07491	−4.2	8.21 (0.52)	5.42 (0.69)	−1.5	0.016	0.045
Creatine	0.716	C_4_H_9_N_3_O_2_	[M+NH_4_]^+^	132.07704	132.07727	−1.7	3.54 (0.34)	2.33 (0.34)	−1.5	0.016	0.045
7H-[1,2,4]Triazolo[4,3-b][1,2,4]triazole-3,7-diamine	0.648	C_3_H_5_N_7_	[M+Na]^+^	140.0679	140.06790	0.0	2.85 (1.10)	2.11 (0.11)	−1.5	0.009	0.027
Emetine N-oxide	0.612	C_29_H_40_N_2_O_5_	[M+H]^+^	249.15613	249.15414	8.0	6.46 (0.30)	3.88 (0.40)	−1.7	0.009	0.027
Carbamazepine 10,11-epoxide	1.516	C_15_H_12_N_2_O_2_	[M+H]^+^	236.07085	236.07060	1.1	1.45 (0.06)	0.63 (0.00)	−2.3	0.005	0.017
Leu-Ala	2.480	C_9_H_16_N_2_O_2_	[M+H]^+^	185.12831	185.12840	−0.5	1.04 (0.07)	0.44 (0.00)	−2.4	0.005	0.017
Quinoline	2.561	C_9_H_7_N	[M+H]^+^	130.0654	130.06512	2.2	1.09 (0.04)	0.40 (0.00)	−2.6	0.005	0.017
Spermidine	0.470	C_7_H_19_N_3_	[M+NH_4_]^+^	146.16516	146.16518	−0.1	1.95 (0.46)	0.76 (0.00)	−2.7	0.005	0.017
SPB 19:0;2O	7.998	C_19_H_41_NO_2_	[M+H]^+^	316.32098	316.32101	−0.1	1.06 (0.23)	0.41 (0.00)	−2.8	0.005	0.017
Uric acid	1.064	C_5_H_4_N_4_O_3_	[M+H]^+^	169.03545	169.03560	−0.9	1.45 (0.33)	0.48 (0.00)	−2.8	0.005	0.017
(2E,6E,12E)-19-(2-amino-2-oxoethyl)-9,11-dihydroxy-8-methoxy- 10,12,14-trimethyl-15-oxohenicosa-2,6,12-trienedioic acid	10.604	C_27_H_43_NO_9_	[M+H]^+^	543.32605	543.32800	−3.6	1.15 (0.07)	0.38 (0.00)	−2.9	0.005	0.017
Corticosterone	5.798	C_21_H_30_O_4_	[M+Na]^+^	347.22122	347.22131	−0.3	2.10 (0.25)	0.74 (0.00)	−3.0	0.005	0.017
DL-Octopamine	1.377	C_8_H_11_NO_2_	[M+H-H_2_O]^+^	136.07574	136.07570	0.3	1.36 (0.01)	0.40 (0.00)	−3.3	0.005	0.017
5-S-Methylthioadenosine	2.576	C_11_H_15_N_5_O_3_S	[M+H]^+^	298.09634	298.09683	−1.6	0.95 (0.20)	0.29 (0.00)	−3.7	0.005	0.017
AUDA	5.668	C_23_H_40_N_2_O_3_	[M+H]^+^	216.1956	216.19580	−0.9	1.31 (0.77)	0.28 (0.00)	−4.2	0.005	0.017
(R)-Prunasin	3.286	C_14_H_17_NO_6_	[M+H]^+^	340.10226	340.10379	−4.5	0.83 (0.53)	0.26 (0.00)	−4.3	0.005	0.017
2,2,6,6-Tetramethyl-4-piperidinyl 2-methylacrylate	6.097	C_13_H_23_NO_2_	[M+NH_4_]^+^	226.18021	226.18021	0.0	5.22 (3.65)	0.98 (0.00)	−5.0	0.005	0.017
Leu-Pro	1.997	C_11_H_20_N_2_O_3_	[M+NH_4_]^+^	229.1545	229.15469	−0.8	1.37 (0.15)	0.25 (0.00)	−5.1	0.005	0.017
Jasminoside	6.098	C_15_H_20_O_3_	[M+Na]^+^	266.17273	266.17380	−4.0	3.54 (1.24)	0.59 (0.00)	−5.2	0.005	0.017
Kynurenine	2.096	C_10_H_12_N_2_O_3_	[M+NH_4_]^+^	209.09207	209.09207	0.0	1.50 (0.55)	0.25 (0.00)	−6.0	0.005	0.017
3-(4-hydroxy-3-methoxyphenyl)prop-2-enamide	3.386	C_10_H_11_NO_3_	[M+H]^+^	194.08073	194.08099	−1.3	1.11 (0.39)	0.19 (0.00)	−6.3	0.005	0.017
Diisooctyl phthalate	12.351	C_24_H_38_O_4_	[M+Na]^+^	408.30878	408.31079	−4.9	7.37 (3.06)	1.09 (0.00)	−6.3	0.005	0.017
2-(1′,2′,3′,4′-Tetrahydroxybutyl)quinoxaline	3.585	C_12_H_14_N_2_O_4_	[M+H]^+^	251.10272	251.10260	0.5	1.66 (0.56)	0.15 (0.00)	−10.1	0.005	0.017
Spiroxamine	9.949	C_18_H_35_NO_2_	[M+H]^+^	298.27356	298.27399	−1.4	1.60 (1.24)	0.12 (0.00)	−10.6	0.005	0.017
Cer 8:0;2O/14:0	9.918	C_22_H_45_NO_3_	[M+H]^+^	372.34747	372.34723	0.6	1.18 (0.79)	0.10 (0.00)	−14.5	0.005	0.017
1-(Cyclohexylmethyl)proline	5.505	C_12_H_21_NO_2_	[M+NH_4_]^+^	212.16447	212.16451	−0.2	1.75 (2.03)	0.11 (0.00)	−17.6	0.005	0.017
Sydonic acid	4.149	C_15_H_22_O_4_	[M+NH_4_]^+^	266.15985	266.16000	−0.6	1.91 (0.45)	0.09 (0.00)	−19.7	0.005	0.017
Adenine	4.149	C_5_H_5_N_5_	[M+Na]^+^	271.11545	271.11630	−3.1	1.74 (0.41)	0.08 (0.00)	−19.9	0.005	0.017
Icaridin	5.506	C_12_H_23_NO_3_	[M+H]^+^	230.17514	230.17509	0.2	3.79 (4.19)	0.22 (0.00)	−19.9	0.007	0.023
Pentyl-b-D-glucopyranoside	4.149	C_11_H_22_O_6_	[M+H]^+^	249.13336	249.13440	−4.2	3.14 (1.39)	0.13 (0.00)	−22.0	0.005	0.017
(2R)-*N*-(3-Ethoxypropyl)-2,4-dihydroxy-3,3-dimethylbutanamide	4.880	C_11_H_23_NO_4_	[M+H]^+^	216.15933	216.15939	−0.3	2.01 (2.49)	0.09 (0.00)	−25.7	0.005	0.017
7-Keto-8-aminopelargonic acid	3.563	C_9_H_17_NO_3_	[M+H]^+^	188.12801	188.12810	−0.5	5.46 (7.53)	0.19 (0.00)	−31.8	0.005	0.017
Phosphorylcholine	10.271	C_5_H_14_NO_4_P	[M+H]^+^	184.07349	184.07332	0.9	1.29 (0.09)	0.03 (0.00)	−45.1	0.005	0.017
Phosphocholine	10.093	C_5_H_14_NO_4_P	[M+H]^+^	184.073	184.07300	0.0	1.12 (0.27)	0.03 (0.00)	−48.1	0.005	0.017
Cer 8:1;2O/2:0	4.228	C_10_H_19_NO_3_	[M+H]^+^	202.14378	202.14377	0.0	1.62 (1.81)	0.03 (0.00)	−53.7	0.005	0.017
Triphenylphosphine oxide	6.829	C_18_H_15_OP	[M+H]^+^	279.09348	279.09329	0.7	1.05 (1.97)	0.02 (0.00)	−59.7	0.005	0.017
Cyclo(L-Leu-L-Pip-L-Aoe-D-Phe)	9.824	C_31_H_44_N_4_O_6_	[M+H]^+^	603.29346	603.29547	−3.3	2.34 (0.27)	0.04 (0.00)	−61.3	0.005	0.017
*N*-cis-Hexadec-9-enoyl-L-homoserine lactone	8.009	C_20_H_35_NO_3_	[M+H]^+^	338.26685	338.26901	−6.4	2.42 (2.35)	0.04 (0.00)	−65.0	0.005	0.017
Methyprylon	4.229	C_10_H_17_NO_2_	[M+H]^+^	184.13274	184.13280	−0.3	1.11 (1.51)	0.02 (0.00)	−74.2	0.005	0.017
Melophlin D/H/I/J	7.821	C_20_H_35_NO_3_	[M+Na]^+^	338.26645	338.26700	−1.6	1.43 (1.54)	0.02 (0.00)	−77.6	0.005	0.017
6-Oxooctadecanoic acid	8.010	C_18_H_34_O_3_	[M+H]^+^	316.28479	316.28461	0.6	3.60 (3.53)	0.04 (0.00)	−89.9	0.005	0.017
Palmitoleoyl ethanolamide	9.066	C_18_H_35_NO_2_	[M+NH_4_]^+^	280.26373	280.26349	0.9	5.11 (5.48)	0.05 (0.00)	−95.0	0.005	0.017
*N*-Acetylleucine	2.920	C_8_H_15_NO_3_	[M+H]^+^	174.11209	174.11230	−1.2	2.76 (3.64)	0.02 (0.00)	−120.3	0.005	0.017
PC O-18:1	12.398	C_26_H_52_NO_7_P	[M+H]^+^	522.35602	522.35541	1.2	2.59 (0.86)	0.02 (0.00)	−133.9	0.005	0.017
LPC 18:1	12.397	C_26_H_52_NO_7_P	[M+Na]^+^	544.3385	544.33734	2.1	2.35 (0.56)	0.02 (0.00)	−151.9	0.005	0.017
Linoleoylglycine	8.775	C_20_H_35_NO_3_	[M+Na]^+^	320.25613	320.25839	−7.1	2.48 (2.77)	0.01 (0.00)	−153.7	0.005	0.017
Oleamide	8.775	C_18_H_35_NO	[M+H-H_2_]^+^	280.26425	280.26349	2.7	6.13 (6.71)	0.02 (0.00)	−253.4	0.005	0.017
LPC 18:3-SN1	9.679	C_26_H_48_NO_7_P	[M+H]^+^	518.32361	518.32410	−0.9	0.78 (0.22)	0.00 (0.00)	−262.5	0.005	0.017
1-Myristoyl-sn-glycero-3-phosphocholine	9.571	C_22_H_46_NO_7_P	[M+H]^+^	468.30911	468.30850	1.3	0.88 (0.22)	0.00 (0.00)	−262.8	0.005	0.017
1-Oleoyl-sn-glycero-3-phosphocholine	12.196	C_26_H_52_NO_7_P	[M+H]^+^	522.35565	522.35541	0.5	1.50 (0.18)	0.01 (0.00)	−267.3	0.005	0.017
Neofusapyrone	11.065	C_34_H_54_O_9_	[M+H]^+^	571.35883	571.36292	−7.2	1.19 (0.37)	0.01 (0.00)	−294.0	0.005	0.017
LPC 16:0	11.556	C_24_H_50_NO_7_P	[M+Na]^+^	518.32312	518.32172	2.7	3.45 (2.80)	0.02 (0.00)	−308.4	0.005	0.017
LPC 15:0-SN1	10.589	C_23_H_48_NO_7_P	[M+H]^+^	482.32422	482.32413	0.2	1.14 (0.27)	0.00 (0.00)	−398.0	0.005	0.017
PC O-20:5	10.232	C_28_H_48_NO_7_P	[M+H]^+^	542.32288	542.32410	−2.2	2.89 (0.37)	0.00 (0.00)	−635.1	0.005	0.017

The data represent the median and interquartile range [Q1; Q3] for the basal time point and day 7, with values normalized by dividing by 10,000. The fold change (FC) was calculated as the median of basal values divided by the median of day 7 values. The false discovery rate (FDR) is the adjusted *p*-value obtained from a paired Mann–Whitney test. Retention time (*R_t_*) is reported in minutes. AUDA: 12-[[(tricyclo[1,3,7,13]dec-1-ylamino)carbonyl]amino]-dodecanoic acid; Cer: Ceramide; LPC: Lysophosphatidylcholine; PC O: 1-alkyl, 2-acylglycerophosphocholines; SPB: sphingosines; DGGA: diacylglycerols.

## Data Availability

The original contributions presented in the study are included in the article; further inquiries can be directed to the corresponding authors.

## Supplementary Materials

The following supporting information can be downloaded at https://www.mdpi.com/article/10.3390/nu17050885/s1, Supplementary Table S1. Metabolites identified in cocoa shell extract, their associated metabolic pathways, and potential health effects. Supplementary Table S2. Key metabolites detected in rat plasma after cocoa shell extract supplementation, their associated metabolic pathways, and potential health effects.

## References

[1] Nirmal N.P., Khanashyam A.C., Mundanat A.S., Shah K., Babu K.S., Thorakkattu P., Al-Asmari F., Pandiselvam R. (2023). Valorization of Fruit Waste for Bioactive Compounds and Their Applications in the Food Industry. Foods.

[2] Kumar K., Yadav A.N., Kumar V., Vyas P., Dhaliwal H.S. (2017). Food Waste: A Potential Bioresource for Extraction of Nutraceuticals and Bioactive Compounds. Bioresour. Bioprocess..

[3] Gil-Ramírez A., Cañas S., Cobeta I.M., Rebollo-Hernanz M., Rodríguez-Rodríguez P., Benítez V., Arribas S.M., Martín-Cabrejas M.A., Aguilera Y. (2024). Uncovering Cocoa Shell as a Safe Bioactive Food Ingredient: Nutritional and Toxicological Breakthroughs. Futur. Foods.

[4] Rebollo-Hernanz M., Aguilera Y., Martin-Cabrejas M.A., Gonzalez de Mejia E. (2022). Phytochemicals from the Cocoa Shell Modulate Mitochondrial Function, Lipid and Glucose Metabolism in Hepatocytes via Activation of FGF21/ERK, AKT, and MTOR Pathways. Antioxidants.

[5] Rodríguez-Rodríguez P., Ragusky K., Phuthong S., Ruvira S., Ramiro-Cortijo D., Cañas S., Rebollo-Hernanz M., Morales M.D., López de Pablo Á.L., Martín-Cabrejas M.A. (2022). Vasoactive Properties of a Cocoa Shell Extract: Mechanism of Action and Effect on Endothelial Dysfunction in Aged Rats. Antioxidants.

[6] Sánchez M., Laca A., Laca A., Díaz M. (2023). Cocoa Bean Shell: A By-Product with High Potential for Nutritional and Biotechnological Applications. Antioxidants.

[7] Rojo-Poveda O., Barbosa-Pereira L., Zeppa G., Stévigny C. (2020). Cocoa Bean Shell—A By-Product with Nutritional Properties and Biofunctional Potential. Nutrients.

[8] Rebollo-Hernanz M., Cañas S., Braojos C., Cano-Muñoz P., Martín-Cabrejas M.A., Campos-Vega R., Oomah B.D. (2022). Cocoa Shell: Source of Novel Bioactive Ingredients for the Prevention of Cardiometabolic Diseases. Molecular Mechanisms of Functional Food.

[9] Mozaffarian D. (2020). Dietary and Policy Priorities to Reduce the Global Crises of Obesity and Diabetes. Nat. Food.

[10] Cañas S., Rebollo-Hernanz M., Braojos C., Benítez V., Ferreras-Charro R., Dueñas M., Aguilera Y., Martín-Cabrejas M.A. (2022). Gastrointestinal Fate of Phenolic Compounds and Amino Derivatives from the Cocoa Shell: An in Vitro and in Silico Approach. Food Res. Int..

[11] Muthubharathi B.C., Gowripriya T., Balamurugan K. (2021). Metabolomics: Small Molecules that Matter More. Mol. Omi..

[12] Guasch-Ferre M., Bhupathiraju S.N., Hu F.B. (2018). Use of Metabolomics in Improving Assessment of Dietary Intake. Clin. Chem..

[13] Rafiq T., Azab S.M., Teo K.K., Thabane L., Anand S.S., Morrison K.M., De Souza R.J., Britz-Mckibbin P. (2021). Nutritional Metabolomics and the Classification of Dietary Biomarker Candidates: A Critical Review. Adv. Nutr..

[14] Clarke E.D., Ferguson J., Collins C.E. (2023). Dietary Assessment and Metabolomic Methodologies in Feeding Studies: A Scoping Review. Proc. Nutr. Soc..

[15] Rebollo-Hernanz M., Cañas S., Taladrid D., Segovia Á., Bartolomé B., Aguilera Y., Martín-Cabrejas M.A. (2021). Extraction of Phenolic Compounds from Cocoa Shell: Modeling Using Response Surface Methodology and Artificial Neural Networks. Sep. Purif. Technol..

[16] Ruvira S., Rodríguez-Rodríguez P., Ramiro-Cortijo D., Martín-Trueba M., Martín-Cabrejas M.A., Arribas S.M. (2023). Cocoa Shell Extract Reduces Blood Pressure in Aged Hypertensive Rats via the Cardiovascular Upregulation of Endothelial Nitric Oxide Synthase and Nuclear Factor (Erythroid-Derived 2)-like 2 Protein Expression. Antioxidants.

[17] Chen Y., Li E.M., Xu L.Y. (2022). Guide to Metabolomics Analysis: A Bioinformatics Workflow. Metabolites.

[18] Tiffany C.R., Bäumler A.J. (2019). Omu, a Metabolomics Count Data Analysis Tool for Intuitive Figures and Convenient Metadata Collection. Microbiol. Resour. Announc..

[19] Lê S., Josse J., Husson F. (2008). FactoMineR: An R Package for Multivariate Analysis. J. Stat. Softw..

[20] Wohlgemuth G., Haldiya P.K., Willighagen E., Kind T., Fiehn O. (2010). The Chemical Translation Service-a Web-Based Tool to Improve Standardization of Metabolomic Reports. Bioinformatics.

[21] Picart-Armada S., Fernández-Albert F., Vinaixa M., Yanes O., Perera-Lluna A. (2018). FELLA: An R Package to Enrich Metabolomics Data. BMC Bioinform..

[22] Higashi Y. (2019). Coffee and Endothelial Function: A Coffee Paradox?. Nutrients.

[23] Rodríguez-Artalejo F., López-García E. (2018). Coffee Consumption and Cardiovascular Disease: A Condensed Review of Epidemiological Evidence and Mechanisms. J. Agric. Food Chem..

[24] Borghi C. (2022). Coffee and Blood Pressure: Exciting News!. Blood Press..

[25] So J., Wu D., Lichtenstein A.H., Tai A.K., Matthan N.R., Maddipati K.R., Lamon-Fava S. (2021). EPA and DHA Differentially Modulate Monocyte Inflammatory Response in Subjects with Chronic Inflammation in Part via Plasma Specialized Pro-Resolving Lipid Mediators: A Randomized, Double-Blind, Crossover Study. Atherosclerosis.

[26] Sherratt S.C.R., Libby P., Budoff M.J., Bhatt D.L., Mason R.P. (2023). Role of Omega-3 Fatty Acids in Cardiovascular Disease: The Debate Continues. Curr. Atheroscler. Rep..

[27] Rao A.S., Nair A., Nivetha K., Ayesha B., Hardi K., Divya V., Veena S.M., Anantharaju K.S., More S.S. (2024). Impacts of Omega-3 Fatty Acids, Natural Elixirs for Neuronal Health, on Brain Development and Functions. Methods Mol. Biol..

[28] Hachem M., Nacir H. (2022). Emerging Role of Phospholipids and Lysophospholipids for Improving Brain Docosahexaenoic Acid as Potential Preventive and Therapeutic Strategies for Neurological Diseases. Int. J. Mol. Sci..

[29] Roy P., Tomassoni D., Nittari G., Traini E., Amenta F. (2022). Effects of Choline Containing Phospholipids on the Neurovascular Unit: A Review. Front. Cell. Neurosci..

[30] Xia J.G., Li B., Zhang H., Li Q.X., Lam S.M., Yin C.L., Tian H., Shui G. (2023). Precise Metabolomics Defines Systemic Metabolic Dysregulation Distinct to Acute Myocardial Infarction Associated with Diabetes. Arterioscler. Thromb. Vasc. Biol..

[31] Paapstel K., Kals J., Eha J., Tootsi K., Ottas A., Piir A., Jakobson M., Lieberg J., Zilmer M. (2018). Inverse Relations of Serum Phosphatidylcholines and Lysophosphatidylcholines with Vascular Damage and Heart Rate in Patients with Atherosclerosis. Nutr. Metab. Cardiovasc. Dis..

[32] Geraldo L.H.M., Spohr T.C.L.d.S., Amaral R.F.d., Fonseca A.C.C.d., Garcia C., Mendes F.d.A., Freitas C., dosSantos M.F., Lima F.R.S. (2021). Role of Lysophosphatidic Acid and Its Receptors in Health and Disease: Novel Therapeutic Strategies. Signal Transduct. Target. Ther..

[33] Hao Y., Guo M., Feng Y., Dong Q., Cui M. (2020). Lysophospholipids and Their G-Coupled Protein Signaling in Alzheimer’s Disease: From Physiological Performance to Pathological Impairment. Front. Mol. Neurosci..

[34] Zhu Y., Wei Y.L., Karras I., Cai P.J., Xiao Y.H., Jia C.L., Qian X.L., Zhu S.Y., Zheng L.J., Hu X. (2022). Modulation of the Gut Microbiota and Lipidomic Profiles by Black Chokeberry (*Aronia Melanocarpa* L.) Polyphenols via the Glycerophospholipid Metabolism Signaling Pathway. Front. Nutr..

[35] Shen J., Li X., Zhang X., Li Z., Abulaiti G., Liu Y., Yao J., Zhang P. (2022). Effects of Xinjiang Wild Cherry Plum (Prunus Divaricata Ledeb) Anthocyanin-Rich Extract on the Plasma Metabolome of Atherosclerotic ApoE-Deficient Mice Fed a High-Fat Diet. Front. Nutr..

[36] Gaggini M., Ndreu R., Michelucci E., Rocchiccioli S., Vassalle C. (2022). Ceramides as Mediators of Oxidative Stress and Inflammation in Cardiometabolic Disease. Int. J. Mol. Sci..

[37] Mayorga-Gross A.L., Esquivel P. (2019). Impact of Cocoa Products Intake on Plasma and Urine Metabolites: A Review of Targeted and Non-Targeted Studies in Humans. Nutrients.

[38] Ryan P.J., Riechman S.E., Fluckey J.D., Wu G. (2021). Interorgan Metabolism of Amino Acids in Human Health and Disease. Adv. Exp. Med. Biol..

[39] Vyas C.M., Manson J.A.E., Sesso H.D., Rist P.M., Weinberg A., Kim E., Moorthy M.V., Cook N.R., Okereke O.I. (2024). Effect of Cocoa Extract Supplementation on Cognitive Function: Results from the Clinic Subcohort of the COSMOS Trial. Am. J. Clin. Nutr..

[40] Guerra I.M.S., Ferreira H.B., Melo T., Rocha H., Moreira S., Diogo L., Domingues M.R., Moreira A.S.P. (2022). Mitochondrial Fatty Acid β-Oxidation Disorders: From Disease to Lipidomic Studies—A Critical Review. Int. J. Mol. Sci..

[41] Kazak L., Cohen P. (2020). Creatine Metabolism: Energy Homeostasis, Immunity and Cancer Biology. Nat. Rev. Endocrinol..

[42] Dunwoodie S.L., Bozon K., Szot J.O., Cuny H. (2023). Nicotinamide Adenine Dinucleotide Deficiency and Its Impact on Mammalian Development. Antioxid. Redox Signal..

[43] Huang Z., Xie N., Illes P., Di Virgilio F., Ulrich H., Semyanov A., Verkhratsky A., Sperlagh B., Yu S.G., Huang C. (2021). From Purines to Purinergic Signalling: Molecular Functions and Human Diseases. Signal Transduct. Target. Ther..

[44] Cañas S., Rebollo-Hernanz M., Bermúdez-Gómez P., Rodríguez-Rodríguez P., Braojos C., Gil-Ramírez A., Benítez V., Aguilera Y., Martín-Cabrejas M.A. (2023). Radical Scavenging and Cellular Antioxidant Activity of the Cocoa Shell Phenolic Compounds after Simulated Digestion. Antioxidants.

[45] Braojos C., Rebollo-Hernanz M., Cañas S., Aguilera Y., Gil-Ramírez A., Benítez V., Martín-Cabrejas M.A. (2024). Cocoa Shell Ingredients Improve Their Lipid-Lowering Properties under Simulated Digestion: In Vitro and HepG2 Cells Study. Food Res. Int..

