# Supplementary material for: Untargeted Metabolomics and Chemometrics Elucidate Dynamic Plasma Profile Changes Induced by Cocoa Shell in Female Rats
Source: Nutrients. 2025 Feb 28;17(5):885. doi: 10.3390/nu17050885 (PMC11902091; doi:10.3390/nu17050885)
Supplement: Supplementary file 1 [file nutrients-17-00885-s001.zip › nutrients-3457796-supplementary.pdf]

nutrients

MDPI

Article

Untargeted metabolomics and chemometrics elucidate dynamic plasma profile changes induced by cocoa shell in female rats

David Ramiro-Cortijo [1,2†], Miguel Rebollo-Hernanz [2,3,4†], Pilar Rodriguez-Rodríguez [1,2], Santiago Ruvira [1,2], Silvia M. Arribas [1,2*], Maria A. Martin-Cabrejas [2,3,4*]

1 Department of Physiology, Faculty of Medicine, Universidad Autónoma de Madrid, C/ Arzobispo Morcillo 2, 28029 Madrid, Spain.
2 Food, Oxidative Stress and Cardiovascular Health (FOSCH) Research Group, Universidad Autónoma de Madrid, 28049 Madrid, Spain.
3 Department of Agricultural Chemistry and Food Science, Faculty of Science, C/ Francisco Tomás y Valiente, 7. Universidad Autónoma de Madrid, 28049, Madrid, Spain.
4 Institute of Food Science Research (CIAL, UAM-CSIC). C/ Nicolás Cabrera, 9. Universidad Autónoma de Madrid, 28049, Madrid, Spain.

† Both authors contributed equally and should be considered as first authors.

* Correspondence: silvia.arribas@uam.es (S.M.A.), maria.martin@uam.es (M.A.M.C)

Supplementary Table S1. Metabolites identified in cocoa shell extract, their associated metabolic pathways, and potential health effects.

Metabolite name	Potentially modulated pathway	Health effect	Ref.
Caffeine	Caffeine metabolism	Central nervous system stimulation, increased alertness	doi: 10.7759/cureus.15032
Nicotinoylglycine	Nicotinate and nicotinamide metabolism	Potential role in lipid metabolism and antioxidant defense	doi: 10.1517/13543776.13.10.1513
Choline cation	Phospholipid metabolism	Essential for neurotransmitter synthesis (acetylcholine), liver function, and cell membrane integrity	doi: 10.2174/187152412800792689
Betaine	One-carbon metabolism	Cardiovascular health support, homocysteine regulation, liver function	doi: 10.1515/cclm.2005.187
Phenylalanine	Amino acid metabolism	Precursor to neurotransmitters like dopamine and norepinephrine, mood regulation	doi: 10.1093/jn/137.6.1539s
L-Isoleucine	Branched-chain amino acid metabolism	Muscle protein synthesis, energy production	doi: 10.1017/s0954422423000197
DL-Norvaline	Amino acid metabolism	Potential inhibitor of arginase, may increase nitric oxide levels	doi: 10.1007/s13318-016-0381-y
DL-Norleucine	Amino acid metabolism	Studied as a leucine analog; potential implications in protein synthesis and metabolism.	doi: 10.1007/s10545-012-9524-8
N-Acetyl-L-glutamic acid	Urea cycle	Essential cofactor for carbamoyl phosphate synthetase I; regulates ammonia detoxification	doi: 10.1203/00006450-199004000-00020
3,4-Cyclopropylglutamate	Glutamate pathway	Potential agonist or antagonist effects on glutamate receptors; implications in neurological function	doi: 10.2174/156802611795164990
Stachydrine	Proline betaine metabolism	Potential cardioprotective effects; may influence homocysteine metabolism	doi: 10.1039/d4md00425f
5-oxo-D-Proline	Glutathione metabolism	Intermediate in glutathione metabolism; accumulation associated with metabolic disorders	doi: 10.1038/srep43911
7-Methylxanthine	Purine metabolism	Intermediate in caffeine metabolism; potential renal and skeletal effects	doi: 10.3390/ijms26041510
L-Proline	Amino acid metabolism	Involved in protein synthesis; important for collagen production and wound healing	doi: 10.1007/s00018-019-03363-3
Val-Val	Peptide metabolism	Dipeptide with potential roles in protein metabolism	doi: 10.1002/psc.2637
L-Tyrosine	Amino acid metabolism	Precursor to neurotransmitters like dopamine and norepinephrine; involved in mood regulation	doi: 10.1111/j.1600-0447.2011.01706.x
Leu-Val	Peptide metabolism	Dipeptide with potential roles in protein metabolism	doi: 10.1002/psc.2637
Leu-Ile	Branched-chain amino acid metabolism	Involved in protein synthesis and energy production; may influence muscle metabolism	doi: 10.1002/psc.2637
(Z)-5,8,11-trihydroxyoctadec-9-enoic acid	Fatty acid metabolism	Potential role in inflammatory processes	doi: 10.1002/pca.3322
Piperidine	Alkaloid metabolism	Acts as a building block for various bioactive compounds; may have neuroactive properties.	doi: 10.1038/s41586-023-06716-y
Pro-Val	Dipeptide metabolism	May play a role in protein metabolism; specific health effects not well-documented.	doi: 10.1002/psc.2637

N-Acetylproline	Amino acid derivative metabolism	Potential antioxidant properties; may influence collagen stability.	doi: 10.3390/antiox11091663
1-Palmitoyl-2-oleoylphosphatidylserine	Glycerophospholipid metabolism	Involved in cell membrane structure; may affect cognitive functions.	doi: 10.1529/biophysj.103.029678
L-Pipecolic acid	Lysine degradation pathway	Accumulates in certain metabolic disorders; may have neuromodulatory effects.	doi: 10.1007/s00726-013-1590-1
6-Hydroxynicotinic acid	Nicotinic acid metabolism	Metabolite of nicotine degradation; potential implications in oxidative stress.	doi: 10.1021/acs.biochem.6b00105
trans-2-dodecenedioic acid	Fatty Acid β-Oxidation	Intermediate in fatty acid metabolism; may be involved in energy production.	doi: 10.1007/s11745-016-4174-5
Nicotinic acid	Nicotinate and Nicotinamide Metabolism	Precursor to NAD^+/$NADP^+$; supports energy metabolism and has lipid-lowering effects.	doi: 10.1016/j.metabol.2021.154923
Ile-Phe	Dipeptide metabolism	Limited specific information available; dipeptides can have various bioactive roles.	doi: 10.1002/psc.2637
L-Tryptophan	Tryptophan metabolism	Precursor to serotonin and melatonin; involved in mood regulation and sleep.	doi: 10.1016/b978-0-443-15589-5.00001-3
Ser-Tyr-Lys	Tripeptide metabolism	Limited specific information available; tripeptides can have various bioactive roles.	doi: 10.1002/psc.2637
PE 17:0/22:6	Phospholipid metabolism	Components of cell membranes; may influence membrane fluidity and signaling.	doi: 10.3390/ijms242115693
Procyanidin B2	Flavonoid metabolism	Antioxidant properties; may support cardiovascular health.	doi: 10.1016/j.foodchem.2023.136101
(+)-Catechin	Flavonoid metabolism	Antioxidant properties; may support cardiovascular health.	doi: 10.1016/b978-0-12-398456-2.00021-9
Aurantiamide	Proteasome inhibition	Exhibits anti-inflammatory and anti-tumor properties by inhibiting the proteasome pathway.	doi: 10.3390/cells14030168
1,3,7-Trimethyluric acid	Purine metabolism	Metabolite of caffeine; may influence adenosine receptor activity and has potential antioxidant effects.	doi: 10.2174/97816810844111180401
Gentiobiose	Carbohydrate metabolism	May act as a prebiotic, promoting beneficial gut microbiota.	doi: 10.1016/j.jff.2020.103838
Phenylethylamine	Trace amine-associated receptor signaling	Acts as a neuromodulator; associated with mood regulation and cognitive functions.	doi: 10.3389/fnins.2016.00148
2-Aminophenol	Tyrosine metabolism	Intermediate in the degradation of tyrosine; potential antioxidant properties.	doi: 10.7554/elife.58053
Dianthoside	Flavonoid glycoside metabolism	Exhibits antioxidant and potential anti-inflammatory properties; specific effects in humans or rats are under investigation.	doi: 10.1177/0976500x241309
L-Leucine	Branched-chain amino acid metabolism	Essential amino acid that stimulates muscle protein synthesis and may modulate lipid metabolism.	doi: 10.3390/nu12051299
Trigonelline	Nicotinate and Nicotinamide Metabolism	Acts as a precursor to NAD^+; supplementation has been shown to improve muscle function and mitochondrial health.	doi: 10.1038/s42255-024-00997-x
3,7-Dihydroxyflavone	Flavonoid metabolism	Possesses neuroprotective and antioxidant activities; may influence cognitive functions.	doi: 10.2174/1389557519666190617150051
cAMP	cAMP Signaling pathway	Serves as a secondary messenger in various biological processes, including regulation of metabolism and gene expression.	doi: 10.1016/j.tips.2017.11.006
Ferulamide	Phenylpropanoid metabolism	Exhibits antioxidant properties; potential benefits in reducing oxidative stress-related damage.	doi: 10.3390/compounds4040044
Pro-Ile	Dipeptide metabolism	Limited specific information available; dipeptides can have various bioactive roles.	doi: 10.1002/psc.2637

PC 8:0/30:7	Phospholipid metabolism	Component of cell membranes; may influence membrane fluidity and signaling.	doi: 10.3390/ijms242115693
Pantothenic acid	Coenzyme a biosynthesis	Essential for fatty acid metabolism and energy production; deficiency can lead to metabolic disorders.	doi: 10.1016/b978-0-12-804572-5.00035-5
1,2-Dipalmitoyl-sn-glycero-3-phospho-(1'-rac-glycerol)	Glycerophospholipid metabolism	Major component of pulmonary surfactant; critical for proper lung function.	doi: 10.1016/j.bbalip.2012.09.010
5-Aminovaleric acid	Lysine degradation pathway	Intermediate in lysine metabolism; accumulation may be associated with metabolic disorders.	doi: 10.1016/j.tem.2022.04.004
MGDG O-8:0/12:0	Glycerolipid metabolism	Component of cell membranes; specific health effects not well-documented.	doi: 10.3390/ijms242115693
Val-Phe	Dipeptide metabolism	Limited specific information available; dipeptides can have various bioactive roles.	doi: 10.1002/psc.2637
Theobromine	Purine alkaloid metabolism	Mild stimulant; vasodilation and diuretic effects; may support cardiovascular health.	doi: 10.1007/978-3-642-13443-2_7
4-Acetamidobutanoic acid	Amino acid metabolism	Limited specific information available; potential intermediate in amino acid catabolism.	doi: 10.3390/nu15153378
3-Hydroxy-2-methylpyridine	Pyridine metabolism	Limited specific information available; potential intermediate in vitamin B6 metabolism.	doi: 10.1016/j.ccr.2012.07.001
2,6-Dihydroxypyridine	Pyridine metabolism	Limited specific information available; potential intermediate in the degradation of certain alkaloids.	doi: 10.1007/978-981-13-7462-3_1
4-Hydroxyquinoline	Quinoline metabolism	Limited specific information available; potential antimicrobial properties.	doi: 10.3390/molecules25235689
Citric acid	Tricarboxylic acid (TCA) cycle	Central to energy production; involved in metabolic pathways like glycolysis and fatty acid synthesis.	doi: 10.1007/978-981-16-0723-3_12
Glu-Ile-Arg	Tripeptide metabolism	Limited specific information available; tripeptides can have various bioactive roles.	doi: 10.1002/psc.2637
2-Aminobenzoic acid	Tryptophan metabolism	Precursor to various bioactive compounds; may have anti-inflammatory properties.	doi: 10.3390/su16073052
Guanosine	Purine metabolism	Involved in nucleic acid synthesis; plays a role in cellular signaling and energy transfer.	doi: 10.1007/s00294-019-00963-1
4-Hydroxyphenethylamine	Trace amine-associated receptor signaling	Acts as a neuromodulator; associated with mood regulation and cognitive functions.	doi: 10.1016/j.biopha.2016.07.002
(−)-Epicatechin	Flavonoid metabolism	Exhibits antioxidant properties; may support cardiovascular health and improve insulin sensitivity.	doi: 10.1080/10408398.2020.1723057
3,4,5-Trihydroxyphenethylamine	Catecholamine biosynthesis	Precursor to neurotransmitters like dopamine; influences mood and motor control.	doi: 10.3389/fneur.2021.616820
Sorbicillin	Polyketide metabolism	Exhibits antimicrobial and antifungal properties; potential therapeutic applications.	doi: 10.3390/jof8010062
Ala-Ile	Dipeptide metabolism	May have antioxidant activity; specific health effects not well-documented.	doi: 10.1002/psc.2637
3-Hydroxypicolinic acid	Nicotinate and Nicotinamide Metabolism	Intermediate in NAD^+ biosynthesis; plays a role in cellular energy metabolism.	doi: 10.1038/s41580-020-00313-x
p-Carboxymethylphenylalanine	Amino acid metabolism	Limited information available; potential derivative of phenylalanine with unknown health effects.	doi: 10.1038/labinvest.2017.126
(9Z,12E)-15,16-dihydroxyoctadeca-9,12-dienoic acid	Linoleic acid metabolism	May have anti-inflammatory properties; role in lipid signaling pathways.	doi: 10.3945/an.114.006940
N-Isovaleroylglycine	Leucine metabolism	Limited specific information available; potential metabolite of leucine catabolism.	doi: 10.1373/clinchem.2005.048421

(+)-Cathinone	Monoamine neurotransmitter release	Acts as a stimulant; increases release of dopamine and norepinephrine; potential for abuse and adverse cardiovascular effects.	doi: 10.1016/j.ejphar.2020.173012
N-Acetyl-β-D-mannosamine	Sialic acid biosynthesis	Precursor in the biosynthesis of sialic acids; may influence cell signaling and microbial interactions.	doi: 10.1093/glycob/cwl075
Kynurenic acid	Tryptophan metabolism	Acts as a neuroprotective agent; antagonist at NMDA and α7-nicotinic receptors; elevated levels associated with cognitive impairments.	doi: 10.1111/jnc.14907
Pro-Trp	Dipeptide metabolism	Limited specific information available; dipeptides can have various bioactive roles.	doi: 10.1002/psc.2637
Anabasamine	Alkaloid metabolism	Limited specific information available; potential bioactive compound with unknown health effects.	doi: 10.1016/j.bmc.2020.115820
MGDG 2:0/3:0	Glycerolipid metabolism	Component of cell membranes; specific health effects not well-documented.	doi: 10.1007/978-90-481-8531-3_13
Harmine	β-Carboline alkaloid metabolism	Inhibits monoamine oxidase A; potential antidepressant effects; may promote neurogenesis.	doi: 10.7150/ijbs.23247
N-cis-p-Coumaroyltyrosine	Phenylpropanoid and Tyrosine Metabolism	Limited specific information available; potential antioxidant properties due to its phenolic structure.	doi: 10.1016/j.biopha.2020.110762
Guanine	Purine metabolism	Essential component of nucleic acids; involved in cellular energy transfer and signaling.	doi: 10.1038/s41392-021-00553-z
SM (34:1;O2)	Sphingolipid metabolism	Component of cell membranes; involved in signal transduction and cell recognition.	doi: 10.1089/ars.2011.3940
Kavain	Gabaergic and Voltage-Gated Ion Channel Modulation	Exhibits anxiolytic and sedative effects; may influence mood and cognitive functions.	doi: 10.1016/b978-0-323-85852-6.00030-5
Jaeschkeanadiol	Diterpenoid metabolism	Limited specific information available; potential anti-inflammatory and antimicrobial properties.	doi: 10.1016/j.bcp.2012.07.021
Sclareol	Diterpene metabolism	Exhibits antimicrobial and anti-inflammatory properties; potential therapeutic applications.	doi: 10.3389/fphar.2022.1014105
Cyclo(Leu-Pro)	Cyclic dipeptide metabolism	Displays antimicrobial and anticancer activities; may modulate immune responses.	doi: 10.3390/biom11101515
Tyr-Gly-Gly-Phe-Leu	Neuropeptide metabolism	Fragment of enkephalins; involved in pain modulation and stress responses.	doi: 10.1002/med.21323
Hypoxanthine	Purine metabolism	Intermediate in purine degradation; elevated levels may indicate tissue hypoxia or gout.	doi: 10.3390/ijms24087027
Val-Tyr	Dipeptide metabolism	Limited specific information available; dipeptides can have various bioactive roles.	doi: 10.1002/psc.2637
Leu-Gly	Dipeptide metabolism	Limited specific information available; dipeptides can have various bioactive roles.	doi: 10.1002/psc.2637
L-Saccharopine	Lysine degradation pathway	Intermediate in lysine metabolism; accumulation may be associated with metabolic disorders.	doi: 10.1152/physiolgenomics.00061.2023
Phe-Gly	Dipeptide metabolism	Limited specific information available; dipeptides can have various bioactive roles.	doi: 10.1002/psc.2637
L-Carnitine	Fatty acid oxidation	Facilitates the transport of fatty acids into mitochondria for β-oxidation; supports energy metabolism.	doi: 10.1016/j.bbamcr.2016.01.023
Ala-Val	Dipeptide metabolism	Limited specific information available; dipeptides can have various bioactive roles.	doi: 10.1002/psc.2637

Arg-Gln	Dipeptide metabolism	Limited specific information available; dipeptides can have various bioactive roles.	doi: 10.1002/psc.2637
Phe-Pro	Dipeptide metabolism	Limited specific information available; dipeptides can have various bioactive roles.	doi: 10.1002/psc.2637
Phytosphingosine	Sphingolipid metabolism	Component of cell membranes; may have antimicrobial and anti-inflammatory properties.	doi: 10.1016/j.plipres.2019.100988
5-(Carbamoylamino)pentanoic acid	Amino acid metabolism	Limited specific information available; potential intermediate in amino acid catabolism.	doi: 10.3389/fbioe.2020.00887
Ala-Nle	Dipeptide metabolism	Limited specific information available; dipeptides can have various bioactive roles.	doi: 10.1002/psc.2637
N-Acetylleucine	Amino acid metabolism	May enhance neuronal function; used in the treatment of cerebellar ataxia.	doi: 10.2174/1570159x16666180905093535
PC(16:0-18:0)	Phospholipid metabolism	Major component of cell membranes; involved in lipid signaling.	doi: 10.1152/advan.00088.2006
Ile-Ala	Dipeptide metabolism	Limited specific information available; dipeptides can have various bioactive roles.	doi: 10.1002/psc.2637
N-Acetyltyramine	Phenylethylamine metabolism	Potential neuromodulatory effects; may influence mood and cognition.	doi: 10.1176/jnp.7.1.6
3-Amino-L-tyrosine	Amino acid metabolism	Limited specific information available; potential role in neurotransmitter synthesis.	doi: 10.1016/j.abb.2010.12.017
L-Cysteine	Sulfur amino acid metabolism	Precursor to glutathione; supports antioxidant defenses and detoxification.	doi: 10.1155/2017/9584932
PyroGlu-Val	Dipeptide metabolism	Limited specific information available; dipeptides can have various bioactive roles.	doi: 10.1002/psc.2637
γ-Glu-Glu	Dipeptide metabolism	Limited specific information available; dipeptides can have various bioactive roles.	doi: 10.1002/psc.2637
γ-Glu-Phe	Dipeptide metabolism	Limited specific information available; dipeptides can have various bioactive roles.	doi: 10.1002/psc.2637
N-Acetylphenylalanine	Amino acid metabolism	Limited specific information available; potential role in protein synthesis.	doi: 10.3389/fmolb.2021.801749
6-Oxooctadecanoic acid	Fatty acid metabolism	Limited specific information available; potential role in lipid metabolism.	doi: 10.1021/acs.jmedchem.0c02058
Ile-Ser	Dipeptide metabolism	Limited specific information available; dipeptides can have various bioactive roles.	doi: 10.1002/psc.2637
Leu-Glu	Dipeptide metabolism	Limited specific information available; dipeptides can have various bioactive roles.	doi: 10.1002/psc.2637
L-Homoserine	Amino acid metabolism	Intermediate in the biosynthesis of threonine, methionine, and isoleucine; accumulation may be associated with metabolic disorders.	doi: 10.1038/s41392-023-01569-3
3-Methyladipic acid	Lysine and Tryptophan Metabolism	Byproduct of lysine and tryptophan catabolism; elevated levels may indicate metabolic disorders such as glutaric aciduria.	doi: 10.1007/s10545-011-9289-5
N-Acetyl-DL-valine	Amino acid metabolism	Derivative of valine; potential role in protein synthesis and metabolism.	doi: 10.3390/nu15010068
Tazettine	Alkaloid metabolism	Exhibits potential antitumor and antiviral properties	doi: 10.1016/j.sajb.2020.07.002
Phe-Ile	Dipeptide metabolism	Limited specific information available; dipeptides can have various bioactive roles.	doi: 10.1002/psc.2637
Ser-Leu	Dipeptide metabolism	Limited specific information available; dipeptides can have various bioactive roles.	doi: 10.1002/psc.2637
Leu-Val-Pro	Tripeptide metabolism	Limited specific information available; tripeptides can have various bioactive roles.	doi: 10.1002/psc.2637
L-Homocitrulline	Amino acid metabolism	Intermediate in the urea cycle and lysine degradation; elevated levels may be associated with urea cycle disorders.	doi: 10.3390/biom13050854

Ethyl L-leucinate	Amino acid ester metabolism	Ester derivative of leucine; potential use in prodrug formulations to enhance bioavailability.	doi: 10.3390/molecules23092318
Linoleoyl ethanolamide	Endocannabinoid metabolism	Modulates appetite, inflammation, and pain perception; acts as an endocannabinoid receptor agonist.	doi: 10.1016/j.plipres.2022.101194
PC (16:0/22:5)	Phospholipid metabolism	Component of cell membranes; may influence membrane fluidity and signaling.	doi: 10.1194/jlr.m037622
Ala-Phe	Dipeptide metabolism	Limited specific information available; dipeptides can have various bioactive roles.	doi: 10.1002/psc.2637
trans-Cinnamic acid	Phenylpropanoid pathway	Exhibits antioxidant and anti-inflammatory properties; may support metabolic health.	doi: 10.3390/nu9020163
5-Methoxytryptophan	Tryptophan metabolism	Limited specific information available; potential neuromodulatory effects.	doi: 10.1002/psc.2637
His-Thr-Lys	Tripeptide metabolism	Limited specific information available; tripeptides can have various bioactive roles.	doi: 10.1038/srep25374
Scalusamide A	Secondary metabolite pathways	Limited specific information available; potential antimicrobial properties.	doi: 10.1002/psc.2637
Lys-Asn	Dipeptide metabolism	Limited specific information available; dipeptides can have various bioactive roles.	doi: 10.1002/psc.2637
Glu-Pro	Dipeptide metabolism	Limited specific information available; dipeptides can have various bioactive roles.	doi: 10.1002/psc.2637
His-Pro	Dipeptide metabolism	Limited specific information available; dipeptides can have various bioactive roles.	doi: 10.1016/j.drudis.2010.06.002
Desmotroposantonin	Sesquiterpene lactone metabolism	Limited specific information available; sesquiterpene lactones are known for anti-inflammatory and anticancer properties.	doi: 10.1002/psc.2637
γ-Glu-Val	Dipeptide metabolism	Limited specific information available; dipeptides can have various bioactive roles.	doi: 10.1007/978-90-481-8531-3_13
MGDG O-8:0/2:0	Glycerolipid metabolism	Limited specific information available; monogalactosyldiacylglycerols (MGDGs) are components of cell membranes, particularly in plants; their effects in humans or rats are not well-documented.	doi: 10.1196/annals.1320.007
L-Propionylcarnitine	Fatty acid oxidation	Facilitates the transport of fatty acids into mitochondria for β-oxidation; may improve exercise performance and alleviate symptoms of peripheral arterial disease.	doi: 10.1093/nar/4.3.739
N6-Succinyladenosine	Purine metabolism	Limited specific information available; potential intermediate in purine metabolism with unknown health effects.	doi: 10.3390/nu13103311
L-Citrulline	Urea cycle	Involved in the detoxification of ammonia via the urea cycle; supplementation may improve exercise performance and cardiovascular health by increasing nitric oxide production.	doi: 10.1002/psc.2637
Phe-Ala-Lys	Tripeptide metabolism	Limited specific information available; tripeptides can have various bioactive roles.	doi: 10.1074/jbc.273.15.8882
N5-(1-Iminoethyl)-L-ornithine	Nitric oxide synthesis inhibition	Inhibits nitric oxide synthase; potential therapeutic applications in conditions with excessive nitric oxide production.	doi: 10.1074/jbc.m604477200
7-Keto-8-aminopelargonic acid	Biotin biosynthesis	Intermediate in the biotin biosynthesis pathway; essential for fatty acid synthesis and energy metabolism.	doi: 10.3389/fphys.2021.702826
4-Methyl-2-oxovaleric acid	Branched-chain amino acid metabolism	Metabolite in isoleucine degradation; elevated levels may indicate metabolic disorders.	doi: 10.1002/psc.2637

Val-Trp	Dipeptide metabolism	Limited specific information available; dipeptides can have various bioactive roles.	doi: 10.1002/psc.2637
Glu-Ala-Lys	Tripeptide metabolism	Limited specific information available; tripeptides can have various bioactive roles.	doi: 10.1021/cb5000532
O-Phospho-L-threonine	Amino acid metabolism	Intermediate in threonine metabolism; involved in protein phosphorylation processes.	doi: 10.1093/glycob/10.1.11
N-Acetylmannosamine	Sialic acid biosynthesis	Precursor in the synthesis of sialic acids; important for cell signaling and glycoprotein function.	doi: 10.1199/tab.0018
2'-Deoxyuridine	Pyrimidine metabolism	Component of DNA; involved in nucleotide metabolism and DNA repair mechanisms.	doi: 10.1002/psc.2637
Gly-Ile	Dipeptide metabolism	Limited specific information available; dipeptides can have various bioactive roles.	doi: 10.1002/psc.2637
Leu-Leu	Dipeptide metabolism	Limited specific information available; dipeptides can have various bioactive roles.	doi: 10.1194/jlr.m037622

SM: Sphingomyelin; PC: Phosphatidylcholine; MGDG: Monogalactosyldiacylglycerol; PE: Phosphatidylethanolamine; cAMP: Cyclic adenosine monophosphate.

Supplementary Table S2. Key metabolites detected in rat plasma after cocoa shell extract supplementation, their associated metabolic pathways, and potential health effects.

**Metabolite name**	**Potentially modulated pathway**	**Health effect**	**Ref.**
1-(4-methylsulfanylphenyl)-3-phenylurea	Prostaglandin synthase	Anti-inflammatory and anticonvulsant	doi: 10.1016/j.ejmech.2018.04.007
LPC O-13:1	Lysophosphatidylcholine metabolism	Involved in inflammatory responses	doi: 10.1182/blood-2008-04-149831
1-(2-Hydroxyethyl)-2,2,6,6-tetramethyl-4-piperidinol	Antioxidant pathways	Potential antioxidant properties	doi: 10.2174/09298671079320539
1,2,3,4-Tetrahydro-b-carboline	Monoamine oxidase inhibition	Neuroprotective effect	doi: 10.1038/sj.bjp.0705653
PC (18:0/22:6)	Phosphatidylcholine metabolism	Supports brain health	doi: 10.1016/j.plipres.2014.06.00
D-erythro-*N*-stearoylsphingosine	Sphingolipid metabolism	Regulates cell growth and apoptosis	doi: 10.1194/jlr.r031278
Glucose	Glycolysis and energy production	Primary energy source	doi: 10.3390/ijms21207729
Docosahexaenoic acid methyl ester	Omega-3 fatty acid metabolism	Supports cardiovascular and brain health	doi: 10.1016/j.eclinm.2021.10099
Theobromine	Caffeine metabolism	Central nervous system mild stimulation	doi: 10.1016/j.jff.2024.106126
N-Isovaleroylglycine	Leucine catabolism	Biomarker for metabolic disorders	doi: 10.1016/j.abst.2023.08.001
Caffeine	Caffeine metabolism	Central nervous system stimulation	doi: 10.7759/cureus.15032
Choline cation	Acetylcholine synthesis	Supports cognitive function	doi: 10.3945/ajcn.110.008938
Cytidine	Nucleotide metabolism	Supports RNA and DNA synthesis	doi: 10.1016/0014-4827(60)90054-9
Tyrosine	Catecholamine synthesis	Precursor to dopamine and norepinephrine	doi: 10.1093/jn/137.6.1539s
Asp-Lys	Protein metabolism	Potential nutritional benefits	doi: 10.3389/fnut.2021.815640
Creatine	Energy metabolism	Enhances muscle performance	doi: 10.1016/s0002-8223(97)00189-2
Leu-Ala	Protein metabolism	Potential nutritional benefits	doi: 10.3389/fnut.2021.815640
Quinoline	DNA intercalation	Antimicrobial properties	doi: 10.1007/5584_2019_428
Spermidine	Autophagy induction	Promotes cellular longevity	doi: 10.14336/ad.2021.0603
Uric acid	Antioxidant pathways	Antioxidant properties	doi: 10.1080/15257770802138558
Corticosterone	Glucocorticoid receptor activation	Regulates stress response	doi: 10.1016/j.yfrne.2007.10.002
5-S-Methylthioadenosine	Polyamine biosynthesis	Anti-inflammatory properties	doi: 10.1152/ajpgi.00549.2011
AUDA	Epoxide hydrolase inhibition	Anti-inflammatory and antihypertensive effects	doi: 10.1016/j.bbrc.2014.03.020
Leu-Pro	Protein metabolism	Potential nutritional benefits	doi: 10.3389/fnut.2021.815640
Kynurenine	Tryptophan metabolism	Modulates immune response and neurological functions	doi: 10.3390/ijms22189879
Cer 8:0;2O/14:0	Sphingolipid metabolism	Cell signaling and apoptosis	doi: 10.1134/s0006297906070030
Adenine	Nucleotide metabolism	Essential for DNA and RNA synthesis	doi: 10.1161/01.res.65.3.531
Pentyl-b-D-glucopyranoside	Carbohydrate metabolism	Potential surfactant properties	doi: 10.3390/molecules29102338
7-Keto-8-aminopelargonic acid	Biotin biosynthesis	Intermediate in biotin production	doi: 10.1128/jb.108.3.1135-1140.1971
Phosphorylcholine	Phospholipid metabolism	Component of cell membranes and lipoproteins	doi: 10.3389/fimmu.2022.7686
Phosphocholine	Phospholipid metabolism	Precursor in phosphatidylcholine synthesis	doi: 10.1091/mbc.01-11-0540
Cer 8:1;2O/2:0	Sphingolipid metabolism	Cell signaling and apoptosis	doi: 10.1134/s0006297906070030
6-Oxooctadecanoic acid	Fatty acid metabolism	Intermediate in lipid oxidation	doi: 10.1152/ajplung.00038.2016
Palmitoleoyl ethanolamide	Endocannabinoid signaling	Potential anti-inflammatory effects	doi: 10.5152/eur-jrheum.2017.17025
N-Acetylleucine	Amino acid metabolism	Control of motion in central nervous system	doi: 10.1007/s00415-022-11534-9
PC O-18:1	Phosphatidylcholine metabolism	Component of cell membranes	doi: 10.3389/fimmu.2022.7686

LPC 18:1	Lysophosphatidylcholine metabolism	Involved in inflammatory responses	doi: 10.1182/blood-2008-04-149831
Linoleoylglycine	Fatty acid amide metabolism	Potential signaling molecule	doi: 10.1016/j.bmcl.2010.08.048
Oleamide	Endocannabinoid signaling	Induces sleep and may have behavior-regulating properties	doi: 10.3389/fnmol.2020.00125
LPC 18:3-SN1	Lysophosphatidylcholine metabolism	Involved in inflammatory responses	doi: 10.1182/blood-2008-04-149831
1-Myristoyl-sn-glycero-3-phosphocholine	Phospholipid metabolism	Component of cell membranes	doi: 10.3389/fimmu.2022.7686
1-Oleoyl-sn-glycero-3-phosphocholine	Phospholipid metabolism	Involved in lipid signaling and metabolism	doi: 10.3389/fimmu.2022.7686
LPC 16:0	Lysophosphatidylcholine metabolism	Involved in inflammatory responses	doi: 10.1182/blood-2008-04-149831
LPC 15:0-SN1	Lysophosphatidylcholine metabolism	Involved in inflammatory responses	doi: 10.1182/blood-2008-04-149831
PC O-20:5	Phosphatidylcholine metabolism	Antioxidant and inflammatory response and membrane structure	doi: 10.3389/fimmu.2022.7686

AUDA: 12-[[(tricyclo[3,3,1,13,7]dec-1-ylamino)carbonyl]amino]-dodecanoic acid; Cer: Ceramide; LPC: Lysophosphatidylcholine; PC O: 1-alkyl, 2-acylglycerophosphocholines.
